# Enzyme sequestration by the substrate: An analysis in the deterministic and stochastic domains

**DOI:** 10.1371/journal.pcbi.1006107

**Published:** 2018-05-17

**Authors:** Andreas Petrides, Glenn Vinnicombe

**Affiliations:** Department of Engineering, University of Cambridge, Cambridge, United Kingdom; ETH Zurich, SWITZERLAND

## Abstract

This paper is concerned with the potential multistability of protein concentrations in the cell. That is, situations where one, or a family of, proteins may sit at one of two or more different steady state concentrations in otherwise identical cells, and in spite of them being in the same environment. For models of multisite protein phosphorylation for example, in the presence of excess substrate, it has been shown that the achievable number of stable steady states can increase linearly with the number of phosphosites available. In this paper, we analyse the consequences of adding enzyme docking to these and similar models, with the resultant sequestration of phosphatase and kinase by the fully unphosphorylated and by the fully phosphorylated substrates respectively. In the large molecule numbers limit, where deterministic analysis is applicable, we prove that there are always values for these rates of sequestration which, when exceeded, limit the extent of multistability. For the models considered here, these numbers are much smaller than the affinity of the enzymes to the substrate when it is in a modifiable state. As substrate enzyme-sequestration is increased, we further prove that the number of steady states will inevitably be reduced to one. For smaller molecule numbers a stochastic analysis is more appropriate, where multistability in the large molecule numbers limit can manifest itself as multimodality of the probability distribution; the system spending periods of time in the vicinity of one mode before jumping to another. Here, we find that substrate enzyme sequestration can induce bimodality even in systems where only a single steady state can exist at large numbers. To facilitate this analysis, we develop a weakly chained diagonally dominant M-matrix formulation of the Chemical Master Equation, allowing greater insights in the way particular mechanisms, like enzyme sequestration, can shape probability distributions and therefore exhibit different behaviour across different regimes.

## Author summary

Models of multisite protein phosphorylation have been of great interest to the systems biology community, largely due to their ability to exhibit multistable behaviour. In the presence of excess substrate it has been shown that the number of stable steady states achieved can increase linearly with the number of phosphosites available. In this paper, we provide a quantitative mathematical analysis of the effect that enzyme docking, and the consequent phosphatase and kinase sequestration by the unphosphorylated and the fully phosphorylated substrates respectively, has on a multisite protein phosphorylation system. The analysis is done in both the deterministic and the stochastic domains, for large and small molecule numbers respectively. We prove, by finding sufficient conditions, that in the deterministic domain substrate enzyme-sequestration must inevitably limit the extent of multistability, ultimately to one steady state, even for systems with arbitrary processivity or sequentiality (i.e. where multiple phosphorylations or dephosphorylations can happen per reaction and in any order). In contrast, in the stochastic domain it can provide bimodality even in cases where bistability is not possible for large molecule numbers.

## Introduction

Probably the most studied form of protein modification is protein phosphorylation, the binding of a phosphoryl ($PO_{3}^{-}$) group using a kinase enzyme [1]. This, together with dephosphorylation by a phosphatase enzyme, contributes to the regulation of transcription factors, thus regulating the response of a cell to changes in its environment [2]. Goldbeter and Koshland [3] showed that ultrasensitivity can be obtained where a sigmoidal change is observed in output for a linear change in input. This, coupled with positive feedback, can result in bistability. Positive feedback, which can be exhibited implicitly by different mechanisms, is required for bistability and consequently for multistability [4]. Examples of such mechanisms are several [5–7]. In this paper we focus on multisite protein phosphorylation, a well studied example of such a mechanism [8–16], itself belonging to the greater class of enzyme-sharing schemes (i.e. when different substrates or substrate states share the same enzymes). This mechanism is of interest because of its potential unlimited multistable behaviour [12, 17], which could be beneficial for using information from environmental signals to drive internal cell processes.

In the excess substrate regime, Thomson and Gunawardena [18] showed that the number of stable steady states that can be achieved increases linearly with the number of phosphosites available. This is done by introducing enzyme saturation and competition between the unphosphorylated and phosphorylated substrate forms for interaction with the free kinase and with the free phosphatase [12, 18]. The ability of this form of competition to induce bistability in a distributive kinetic mechanism of the two-site MAPK (Mitogen-activated protein kinase) phosphorylation and dephosphorylation was firstly shown by Kholodenko et al [12, 19, 20].

**Fig 1:**
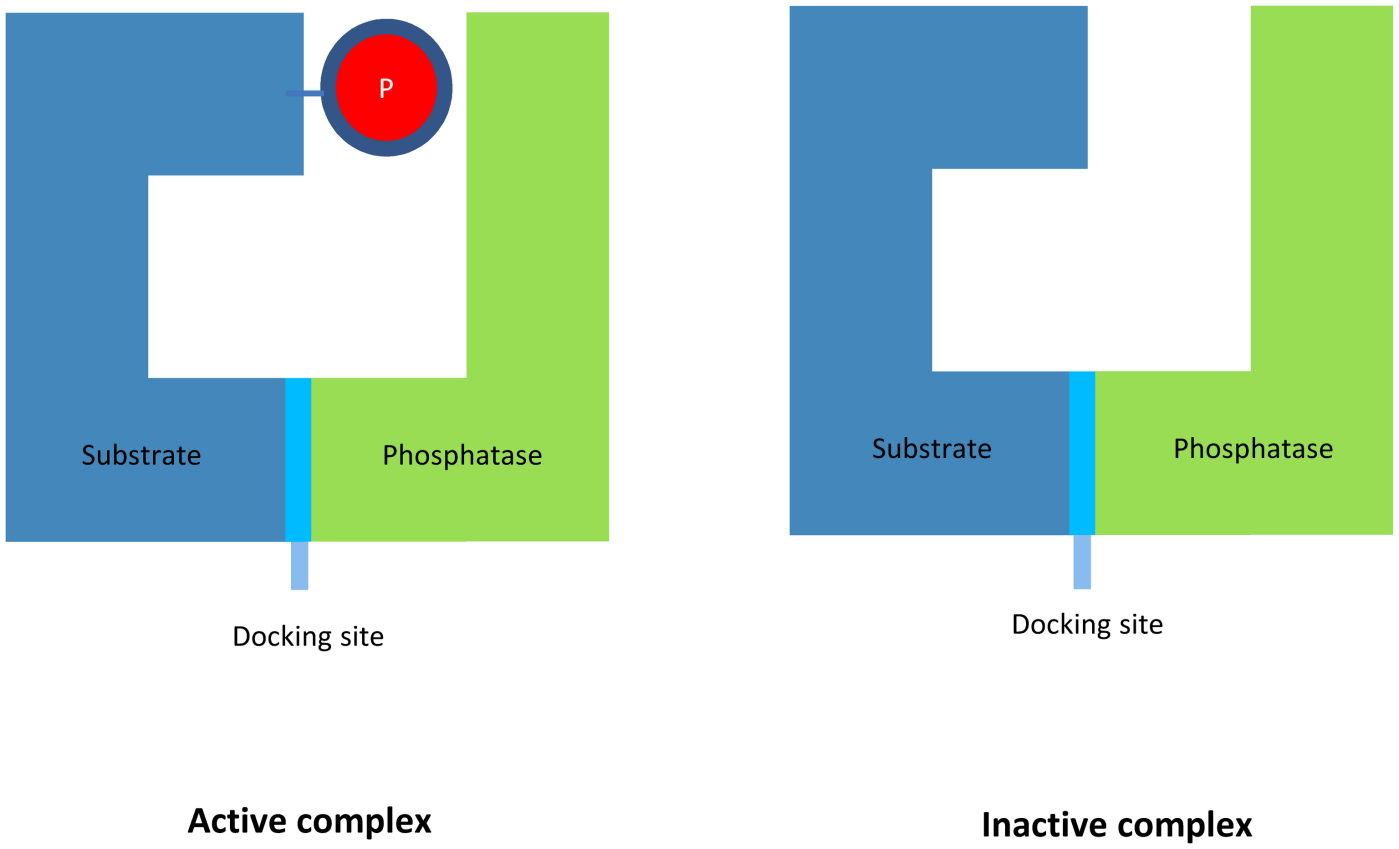
Inactive complex formation. An inactive complex can for example be formed when a phosphatase molecule binds with a completely unphosphorylated substrate.

Nevertheless, it is increasingly being recognised that specificity in protein phosphorylation and dephosphorylation cycles can be achieved through enzyme docking: the binding of the interaction domains on the kinase or phosphatase with one or more docking sites on the substrate, where the latter is separate from the motif that is chemically modified. [11, 21–24]. Examples of such docking interactions that have been identified include MAPK and MAPK phosphatases [25–27], and Glycogen synthase kinase-3 [28], an important kinase for insulin and Wnt signalling [11]. This mechanism implies that a phosphatase molecule can still bind to an unphosphorylated substrate molecule and similarly, a kinase molecule can still bind to a fully-phosphorylated one, forming inactive complexes, as each enzyme can always bind to their docking site [29]. The formation of inactive complexes is graphically illustrated in Fig 1. In the excess substrate regime, the formation of such complexes can be thought of as a sequestration mechanism, where the substrate sequesters away the enzymes. This is referred to in the paper as ‘Substrate Enzyme-Sequestration’. In the complementary regime of excess enzyme, Martins and Swain have already shown that this type of sequestration can provide ultrasensitivity [29].

Note that this mechanism of enzyme sequestration is fundamentally different to that of enzyme sequestration by a different protein (e.g. a scaffold protein) that does not participate in the reaction scheme metabolically [30]. In that type of sequestration, the scaffold-bound population is separated from the rest of the reaction network, creating two compartments. Indeed, compartmentalisation is another mechanism able to provide enhanced ultrasensitivity, bistability and/or multistability [13, 30, 31]. However, in Substrate Enzyme-Sequestration, neither additional proteins nor compartments are sequestering the enzyme; this is done by the substrate itself, as also explained by Martins and Swain [29]. As this sequestration is dependent on the inherent way the substrate attaches to the enzymes, identifying it experimentally is equivalent to identifying whether the enzyme has any means of avoiding the binding with a substrate found in a phosphorylation state which would create an inactive complex, as for example is the binding of a phosphatase to a completely unphosphorylated substrate.

Here we investigate the effect that this type of sequestration can have on multisite protein phosphorylation in the excess substrate regime in the domains of both large and small numbers of molecules, where a deterministic and a stochastic analysis are respectively more suitable. For the stochastic analysis, we develop a new weakly diagonally dominant M-matrix formulation of the Chemical Master Equation, which allows greater insights on the formation of probability distributions, without the necessity of continuously calculating the exact solution of the steady state distribution or running Monte Carlo simulations.

## Models

### A deterministic framework for the excess substrate regime [18]

**Fig 2:**
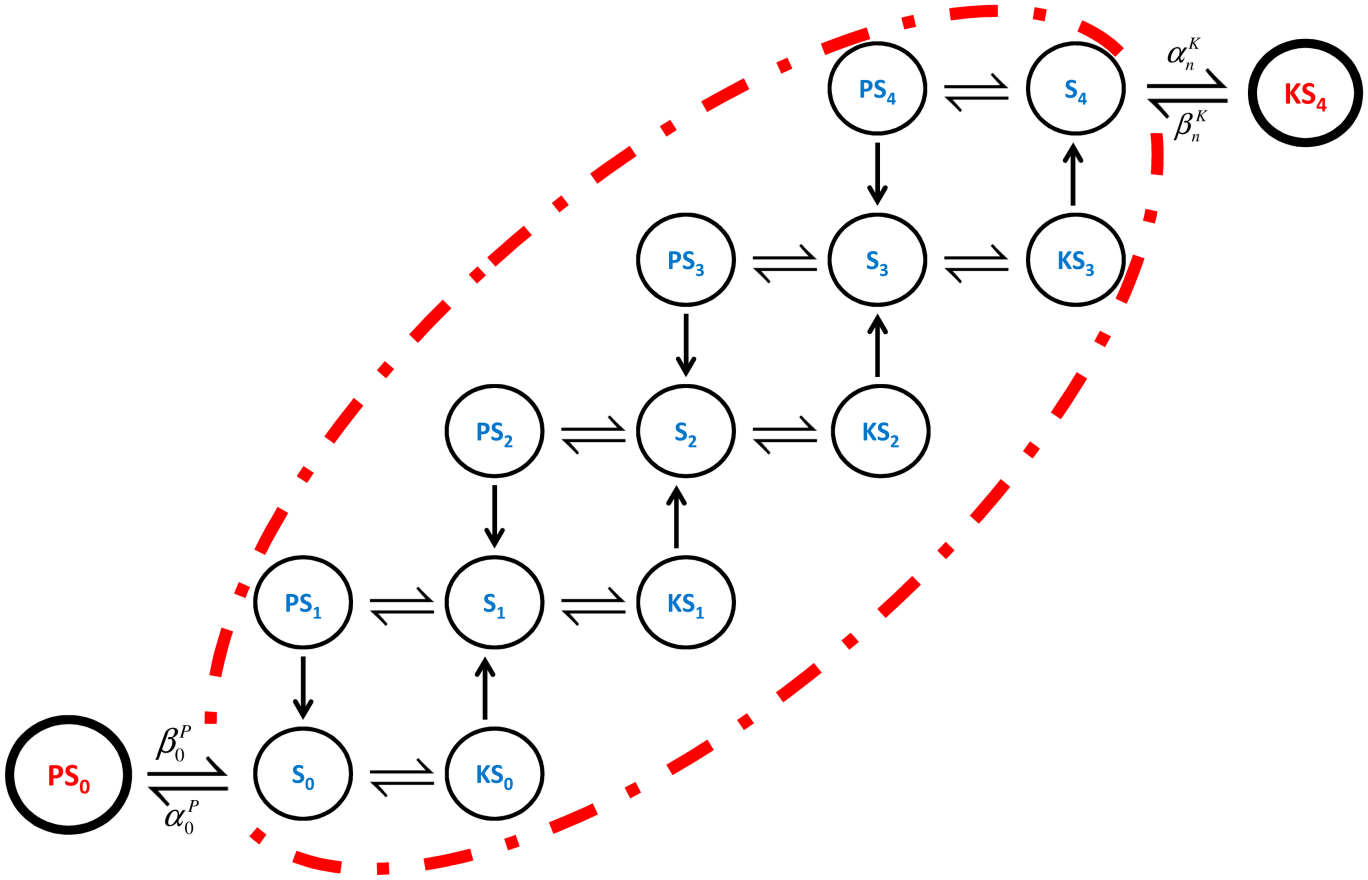
Extension of the 4-site protein phosphorylation scheme. The original 4-site protein phosphorylation scheme is extended to include two inactive complexes *PS*_0_ and *KS*_4_, as implied by enzyme docking.

Our analysis in the large molecule number domain is based on the deterministic framework of Thomson and Gunawardena [18, 32] which was used to show mathematically that unlimited multistability is possible. We first summarise their results, and then extend them to account for Substrate Enzyme-Sequestration by including the reactions outside the red dotted frame of Fig 2. We will show that, irrespective of the other parameters of the system, as the strength of sequestration is increased then the number of steady states decreases to one.

Fig 2 is built from of two kinds of reaction: firstly, a kinase molecule *K* can attach to a substrate molecule with *i* phosphorylated phosphosites, *S*_*i*_. The new complex formed, *KS*_*i*_, can then either decompose back to *K* and *S*_*i*_ or phosphorylation can proceed, leading to the products *K* and *S*_*i*+1_.

$$K+S_{i}\overset{\alpha_{i}^{K}}{\underset{\beta_{i}^{K}}{⇌}}KS_{i}\overset{\gamma_{i}^{K}}{\to }S_{i+1}+K$$

In addition, a phosphatase molecule *P* can attach to a substrate molecule with *i* + 1 phosphorylated phosphosites, *S*_*i*+1_ with the new complex formed *PS*_*i*+1_ either decomposing back to *P* and *S*_*i*+1_ or lead to a dephosphorylation reaction with products *P* and *S*_*i*_.

$$P+S_{i+1}\overset{\alpha_{i+1}^{P}}{\underset{\beta_{i+1}^{P}}{⇌}}PS_{i+1}\overset{\gamma_{i+1}^{P}}{\to }S_{i}+P$$

Using mass action kinetics, the total steady state concentrations of kinase in all its forms, and phosphatase in all its forms, can be written in terms of the concentration of free kinase, free phosphotase and total substrate. Under the assumption of excess substrate, i.e. that the total concentration of substrate [*S*_tot_] ≫ [*K*_tot_] and [*S*_tot_] ≫ [*P*_tot_] then these can be written as

$$\begin{array}{c} \begin{array}{cc} \left[K_{\text{tot}}\right] & =\left[K\right](1+\left[S_{\text{tot}}\right]\frac{\phi_{2}\left(u\right)}{\phi_{1}\left(u\right)})\\ \left[P_{\text{tot}}\right] & =\left[P\right](1+\left[S_{\text{tot}}\right]\frac{\phi_{3}\left(u\right)}{\phi_{1}\left(u\right)}) \end{array} \end{array}$$ (1)

where

$$\begin{array}{l} \left[S_{\text{tot}}\right]=\left[S_{0}\right]+\ldots +\left[S_{n}\right]+\left[KS_{0}\right]+\ldots +\left[KS_{n-1}\right]\\ \begin{array}{cc} & \;+\left[PS_{1}\right]+\ldots +\left[PS_{n}\right] \end{array} \end{array}$$

etc and *ϕ*_1_, *ϕ*_2_, and *ϕ*_3_ are polynomials in *u* = [*K*]/[*P*] of degree *n*. The coefficients of these polynomials can be written in terms of the rate constants *α*, *β* and *γ* in their various subscripted and superscripted forms and, importantly, are all positive (S1 Text, S1.1). Dividing the two expressions and rearranging yields the single polynomial:

$$\begin{array}{c} 0=\left(u-w\right)\phi_{1}+\left[S_{\text{tot}}\right]\left(u\phi_{2}-w\phi_{3}\right)≕P\left(u\right) \end{array}$$

where $w=\frac{\left[K_{\text{tot}}\right]}{\left[P_{\text{tot}}\right]}$. *P*(*u*) is a polynomial in *u* of order *n* + 1.

$$\begin{array}{c} P\left(u\right)=a_{n+1}u^{n+1}+a_{n}u^{n}+\ldots +a_{1}u+a_{0} \end{array}$$ (2)

and the roots of *P*(*u*) correspond to steady state enzyme ratios. Note that these correspond to non-equilibrium steady states of the underlying biochemical system, since each phosphorylation/dephosphorylation cycle is driven in a counter-clockwise direction by the hydrolysis of ATP. Expressions for the *a*_*i*_ in terms of the rate constants are given in S1 Text, Eq. S2. The important point, though, is that the leading coefficient *a*_*n*+1_ is positive, as it derives from the leading coefficients of *ϕ*_1_ and *ϕ*_2_. Conversely, the trailing coefficient *a*_0_ is negative, as it derives from the trailing coefficients of *ϕ*_1_ and *ϕ*_3_ multiplied by −*w* i.e.

$$\begin{array}{c} a_{n+1}>0,\;a_{0}<0 \end{array}$$ (3)

Thus the problem of finding the steady states of the system is transformed into a problem of finding the roots of a univariate polynomial. This polynomial can have no more than *n* + 1 real positive solutions, and Descartes rule of signs was used to show that when *n* is odd there can be no more than *n* real positive solutions (because the reversal of sign between the first and last coefficients limits the number of changes in sign). Thus the maximum number of stable steady states is equal to $1+\lfloor \frac{n}{2}\rfloor$ [18, 32]. Gunawardena and Thomson further showed that it was possible to achieve this number by realistic choices of parameter values. Note however that the extent of multistability observed experimentally is much more limited [12], as also mentioned in their seminal paper [18].

### Substrate-kinase and substrate-phosphatase sequestration

Enzyme docking allows the possibility that an enzyme may attach to substrate even when the complex formed will not be active. Fig 2 represents the reactions occurring when the multisite protein phosphorylation is distributive and sequential as is often taken to be the case [12, 15, 32]. Most of our results generalise to the non-distributive, non-sequential case, but we start by describing the simpler case, where the conclusions are sharper. The inactive complexes formed after a kinase binds to a fully phosphorylated substrate and after a phosphatase binds to an unphosphorylated substrate are shown outside the dashed area. These complexes represent an example of substrate enzyme-sequestration. This occurs when a substrate molecule (e.g. a fully phosphorylated substrate molecule) forms an inactive complex with an enzyme molecule (e.g. a kinase), neither allowing the enzyme to bind to other substrate molecules to form active complexes nor any other enzyme (e.g. a phosphatase) to bind to itself. This effect can occur, for example, through competition of the enzymes for the same, or partly the same, docking sites, as illustrated in literature [26, 33].

#### Model extension with the addition of substrate enzyme sequestration

We now extend the analysis of Gunawardena and Thomson to investigate the potential effects of substrate enzyme-sequestration. First, the polynomials *ϕ* need to be redefined in order to accommodate the sequestration effects, since now conservation of mass includes two extra species, *KS*_*n*_ and *PS*_0_:

$$\begin{array}{cc} {}[S_{\text{tot}}]= & \left[S_{0}\right]+\ldots +\left[S_{n}\right]+\left[KS_{0}\right]+\ldots +\left[KS_{n-1}\right]\\ & +\left[PS_{1}\right]+\ldots +\left[PS_{n}\right]+\left[{\text{PS}}_{0}\right]+\left[{\text{KS}}_{n}\right] \end{array}$$

As shown in Fig 2, each of the two new species interacts with just one of the species of the original system (*S*_*n*_ and *S*_0_). Consequently, at steady state, the flow from *PS*_0_ into the original system via *S*_0_ has to be equal to the flow from the original system to *PS*_0_. Similarly for *S*_*n*_ and *KS*_*n*_.

In order to take these additional species into account, the polynomials *ϕ*_2_ and *ϕ*_3_ need to be modified to include all *n* substrate states in the mass conservation equations. We will show that increasing the strength of sequestration changes only the magnitude of the leading and trailing coefficients in the resulting polynomial *P*(*u*) which, as a further consequence of the sign change in Eq 3, inevitably leads to a reduction in the number of steady states—eventually to one.

## Results/Discussion

### Substrate enzyme-sequestration in the deterministic domain

#### What happens when substrate enzyme-sequestration is considered?

Having extended the deterministic framework to account for substrate enzyme-sequestration, we investigated whether this additional competition for enzymes enhances or inhibits the extent of multistability.

**Fig 3:**
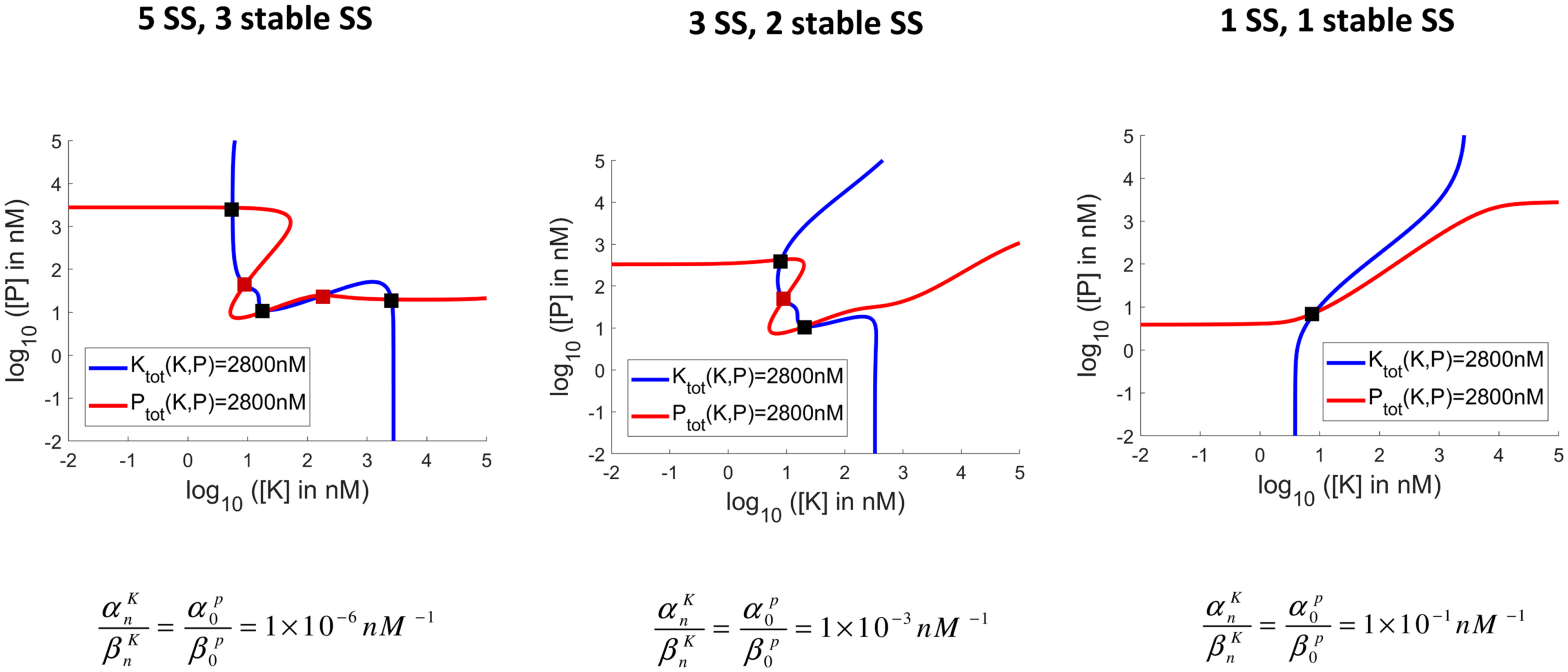
Plots of total concentrations of kinase and phosphatase as functions of free kinase and phosphatase. The intersections correspond to steady states (S1 Text, S1.1). The stable steady states are coloured in black, whereas the unstable steady states are coloured in red. Increasing the strength of both the Substrate Kinase ($\frac{\alpha_{n}^{K}}{\beta_{n}^{K}}$) and Substrate Phosphatase-Sequestration ($\frac{\alpha_{0}^{P}}{\beta_{0}^{P}}$), the number of steady states (and stable steady states) falls in a continuous fashion from 5 (3 stable), as in the system without any sequestration, to 3 (2 stable) and then to 1 (1 stable).

The resulting contours for [*K*_tot_] and [*P*_tot_] are shown in Fig 3, which shows how the original system in [18, 32], with [*K*_tot_] = 2.8*μM*, [*P*_tot_] = 2.8*μM*, [*S*_tot_] = 10*μM* and 4 available phosphosites, is affected as the strength of Substrate Enzyme-Sequestration ($\frac{\alpha_{n}^{K}}{\beta_{n}^{K}}$ and $\frac{\alpha_{0}^{P}}{\beta_{0}^{P}}$) increases. Furthermore, note that the contours presented in the figures represent the accurate non-approximated [*K*_*tot*_] and [*P*_*tot*_] as these are shown in S1 Text, S1.1. As can be seen, as the strength of sequestration is increased the number of steady states (and stable steady states) decreases continuously from 5 (3 of which are stable) in the original tristable system of [18, 32] to 3 (2 of which are stable) to a single stable steady state. Note that the ratios $\frac{\alpha_{i}^{K}}{\beta_{i}^{K}}$ for 0 ≤ *i* < *n* and $\frac{\alpha_{i}^{P}}{\beta_{i}^{P}}$ for 0 < *i* ≤ *n* are approximately equal to 5 × 10^−1^ *nM*^−1^, as seen in S1 Text, S1.11.

#### Sufficient conditions for further limiting the extent of multistability

Having demonstrated the qualitative effect of Substrate Enzyme-Sequestration we now derive quantitative conditions for the reduction in multistability. Repeating the analysis above, with the new polynomials *ϕ*_2_ and *ϕ*_3_ to account for sequestration we obtain a new polynomial, applicable for the regime, Eq 4. Only the first and the last coefficients change compared to Eq 2,.

$$\begin{array}{c} P^{\prime }\left(u\right)=a_{n+1}^{\prime }u^{n+1}+a_{n}u^{n}+\ldots +a_{1}u+a_{0}^{\prime } \end{array}$$ (4)

where

$$\begin{array}{c} \begin{array}{c} a_{n+1}^{\prime }=a{}_{n+1}(1+\left[S_{\text{tot}}\right]\frac{\alpha_{n}^{K}}{\beta_{n}^{K}})\\ a_{0}^{\prime }=a{}_{0}(1+\left[S_{\text{tot}}\right]\frac{\alpha_{0}^{P}}{\beta_{0}^{P}}) \end{array} \end{array}$$

That is, the sign difference between the leading and trailing coefficients is maintained, and they are increased in magnitude. This limits the number of possible positive roots. Using the Vieta formulae, which relate the coefficients of a polynomial to sums and products of its roots [34], in conjunction with the Quadratic Mean—Arithmetic Mean [35] and the Triangle inequalities it is possible to obtain sharp bounds. The following theorem, which is proved in S1 Text, shows that if either the leading three terms or trailing three terms fail a discriminant like condition then the polynomial must have a pair of complex roots, in which case the potential number of real positive roots is reduced by two and the number of stable steady states by one:

**Theorem 1**. *If any of the following conditions are satisfied, then the number of positive steady states will be no more than n* − 1 *if n is even, or n* − 2 *if n is odd:*

1. *a*_*n*−1_ ≤ 0 *and* *a*_2_ ≥ 0
2. *a*_*n*−1_ > 0 *and* $\frac{\alpha_{n}^{K}}{\beta_{n}^{K}}\left[S_{tot}\right]>\frac{na_{n}^{2}-2\left(n+1\right)a_{n+1}a_{n-1}}{2\left(n+1\right)a_{n+1}a_{n-1}}$ *or*
3. *a*_2_ < 0 *and* $\frac{\alpha_{0}^{P}}{\beta_{0}^{P}}\left[S_{tot}\right]>\frac{na_{1}^{2}-2(n+1)a_{0}a_{2}}{2(n+1)a_{0}a_{2}}$

**Fig 4:**
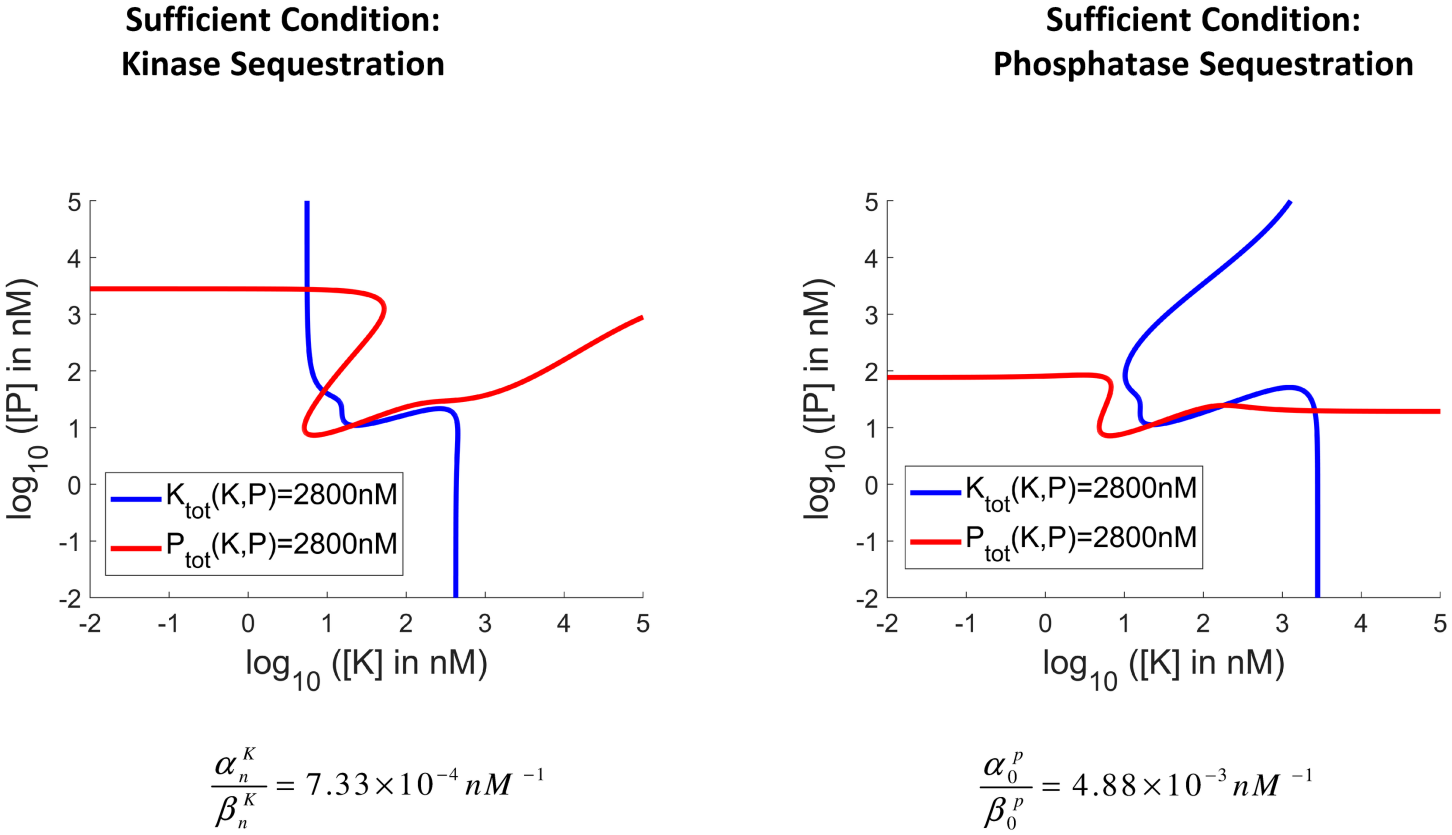
Tightness of the derived sufficient bounds. The sufficient bounds are reasonably tight for the original tristable system for both Substrate Kinase-Sequestration (left) and Substrate Phosphatase-Sequestration. This tightness is portrayed in the figures by the small separation of the *K*_*tot*_ and *P*_*tot*_ contours at the regions where there was previously an intersection.

Thus it is always possible to choose sequestration rates such that the maximum number of stable steady states is equal to $\left\lfloor \frac{n}{2}\right\rfloor$. Fig 4 illustrates that the stated bounds are reasonably tight for the original tristable system [18, 32], as the small gaps between the contours [*K*_tot_] = 2.8*μM* and [*P*_tot_] = 2.8*μM*, in the region of their previous intersections, demonstrate. For the original system to be tristable (i.e. with five steady states) both $\frac{\alpha_{n}^{K}}{\beta_{n}^{K}}\leq 4.4\times 10^{-4}$ *nM*^−1^ and $\frac{\alpha_{0}^{P}}{\beta_{0}^{P}}\leq 3.2\times 10^{-3}$ *nM*^−1^ have to be satisfied as found in simulations. If either condition is violated, then the system becomes bistable. If both are violated, then the system becomes monostable. Our derived conditions are quite close to these, as we find that it is sufficient that $\frac{\alpha_{n}^{K}}{\beta_{n}^{K}}\geq 7.33\times 10^{-4}$ *nM*^−1^ or $\frac{\alpha_{0}^{P}}{\beta_{0}^{P}}\geq 4.88\times 10^{-3}$ *nM*^−1^ is satisfied for tristability to be limited to bistability.

Furthermore, based on the Pratt’s tableau test [36], which is a method of finding a less conservative than Descartes’ rule of signs upper bound for the real positive roots of a real polynomial, the following theorem is proved in S1 Text, S1.4.

**Theorem 2**. *For any δ*^*K*^ ≥ 0 *there exists δ*^*P*^, *directly computable from the rate constants, such that if* $\frac{\alpha_{n}^{K}}{\beta_{n}^{K}}\left[S_{tot}\right]=\delta_{K}$ *and* $\frac{\alpha_{0}^{P}}{\beta_{0}^{P}}\left[S_{tot}\right]\geq \delta^{P}$ *then the polynomial P*′(*u*) *has precisely one positive root, corresponding to one steady state. Similarly, for any δ*^*P*^ ≥ 0 *there exists a δ*_*K*_ *with the same properties*.

As explained in more detail in S1 Text, by setting a *δ*^*K*^, a *δ*^*P*^ can be calculated directly from an algorithm based on the Pratt tableau (which we develop and is found in S1 Text, S1.6) and vice-versa. In this way, we iteratively increased *δ*^*K*^ (the input) until *δ*^*P*^ (the output) could not decrease anymore. This algorithm provided us with the following condition: if $\frac{\alpha_{n}^{K}}{\beta_{n}^{K}}=6.57\times 10^{-4}$ *nM*^−1^ and $\frac{\alpha_{0}^{P}}{\beta_{0}^{P}}\geq 6.928\times 10^{-2}$ *nM*^−1^, then the original tristable system [18, 32] can only have one steady state. Again, these numbers are reasonable when compared to the aforementioned actual values obtained via simulations.

#### Does direct decrease of overall substrate/enzyme numbers have the same effect as substrate enzyme-sequestration?

**Fig 5:**
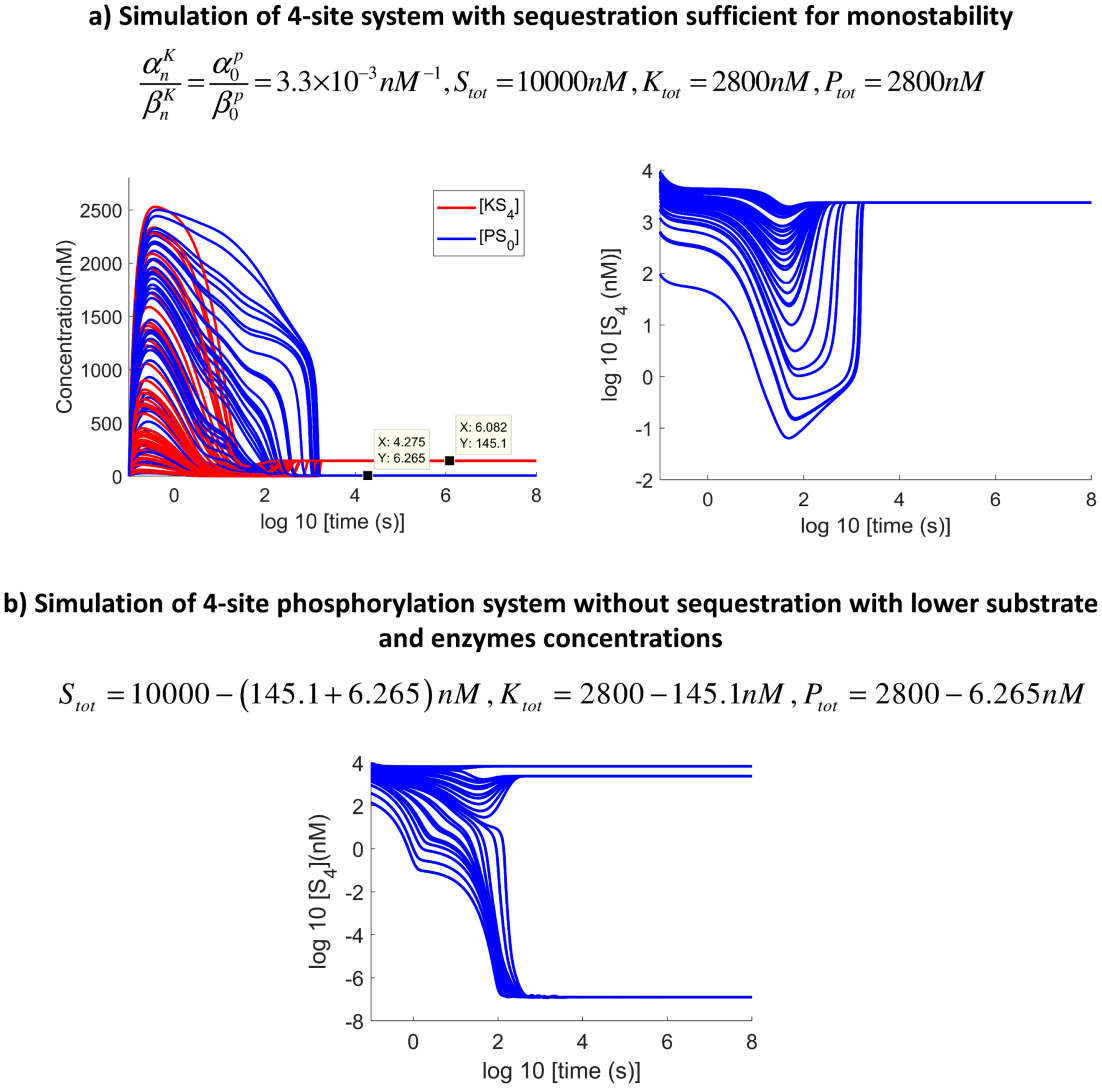
Enzyme sequestration by the substrate does not have the same effect as directly decreasing enzyme and substrate concentrations. a) The 4-site original system simulated with sufficient sequestration for monostability to occur (right) ($\frac{\alpha_{n}^{K}}{\beta_{n}^{K}}=\frac{\alpha_{0}^{P}}{\beta_{0}^{P}}=3.3\times 10^{-3}$ *nM*^−1^). The steady state concentrations of *PS*_0_ and *KS*_4_ due to sequestration are determined (left). b) The same system is simulated with lowering the total substrate and enzyme concentrations by those amounts.

**Fig 6:**
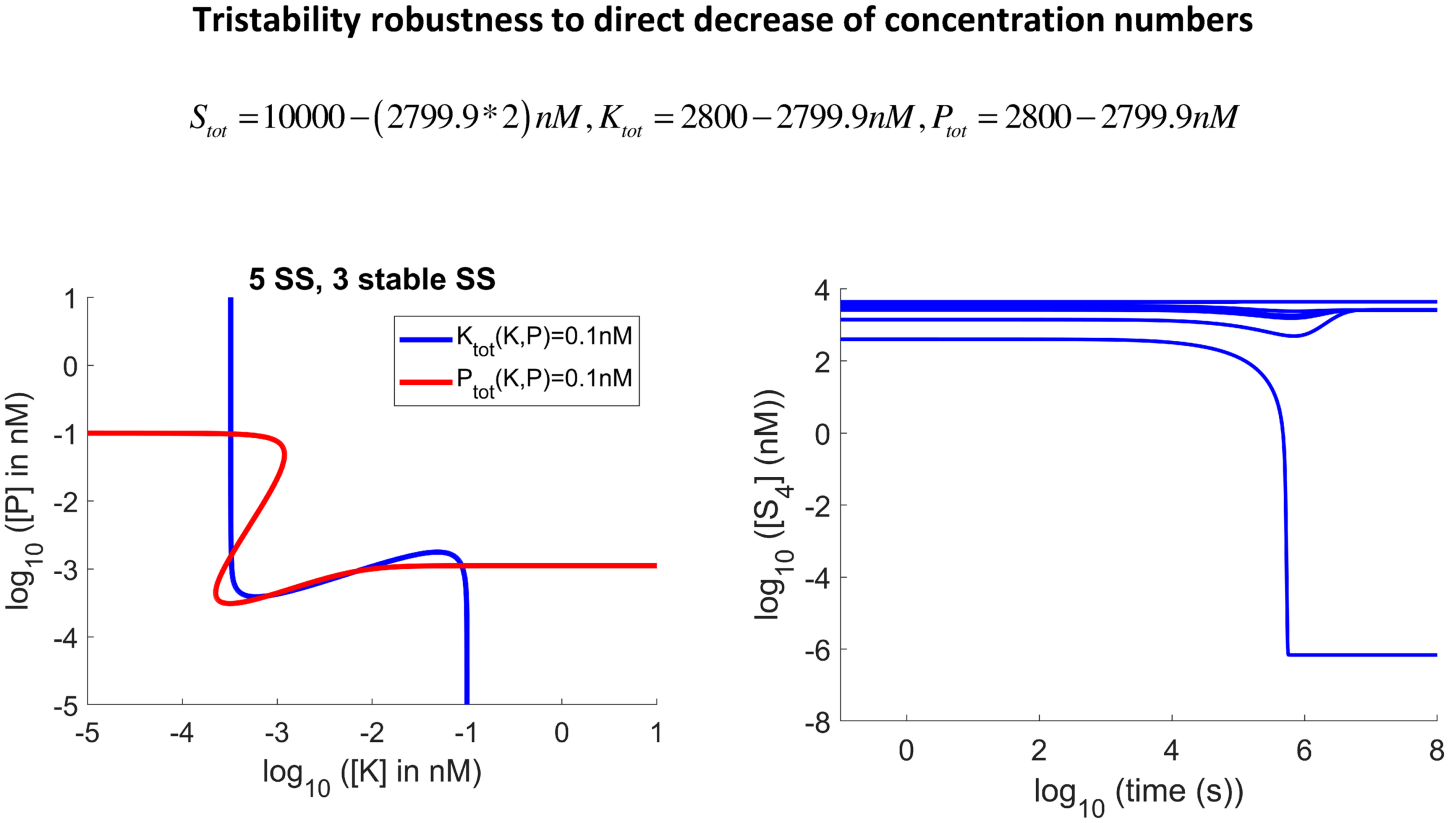
Tristability is very robust to direct decrease of enzyme concentration numbers. Tristability is preserved even when 2799.9*nM* from the 2800*nM* of the total concentration of both the phosphatase and the kinase are removed directly(and therefore 5599.8*nM* from the 10000*nM* of the substrate concentration) without the substrate enzyme-sequestration mechanism.

The inclusion in the model of the inactive complexes *PS*_0_ and *KS*_*n*_ decreases the numbers of both unbound substrate and free enzymes. A natural question then is whether the observed limits on multistability could be attributed simply to these lowered concentrations. In order to investigate this, we found the concentrations of substrate and enzymes that are sequestered away because of the complexes *PS*_0_ and *KS*_*n*_ when $\frac{\alpha_{n}^{K}}{\beta_{n}^{K}}=\frac{\alpha_{0}^{P}}{\beta_{0}^{P}}=3.3\times 10^{-3}$ *nM*^−1^ (i.e. when the system is exhibiting a monostable behaviour). Then we checked the behaviour of the same system without sequestration with the corresponding lower total substrate and enzyme concentrations. We found that substrate enzyme-sequestrations effects cannot be attributed to a simple decrease of the numbers of enzyme and substrate, as the 4-site system still exhibited tristability, as illustrated by Fig 5. In fact, tristability persists even when 2799.9*nM* of the 2800*nM* total concentration of both the phosphatase and the kinase (together with 5599.8*nM* of the 10000*nM* substrate concentration) are removed. This is shown in Fig 6.

#### How does substrate enzyme-sequestration limit multistability?

Having established that it is not the decrease in enzyme concentration numbers that limits multistability we search for an intuitive understanding of what does. It is helpful to consider the simpler two-site, bistable system described by Kholodenko et al [12, 19], which corresponds to the bottom left of Fig 2. Bistability there occurs because the unphosphorylated substrate *S*_0_ inhibits the production of the fully phosphorylated substrate *S*_2_ by competing with the singly-phosphorylated *S*_1_ for the kinase, while *S*_2_ inhibits the production of *S*_0_ by competing with *S*_1_ for the phosphatase [12]. Thus, allowing *S*_0_ to bind with the phosphatase has the effect that it is now inhibiting its own production as well by competing with *S*_1_ for the phosphatase. As it is inhibiting its own production, it now becomes a worse inhibitor for the production of *S*_2_. The same applies to *S*_2_ when the binding with the kinase is permitted. Thus, Substrate Enzyme-Sequestration reduces the coupling which caused bistability in the first place.

This explains our finding that no matter what the other kinetic parameters of the system are, we can always calculate a minimum strength of sequestration which limits the extent of multistability (Theorem 1) or even reduce it to one (Pratt tableau algorithm, S1 Text, S1.6). For the parameters of the model (as in S1 Text, S1.11) i.e. for equal concentrations of kinase and phosphatase (*w* = 1), *S*_*tot*_ large and $\gamma_{0}^{K}<<\gamma_{1}^{P}$, $\gamma_{2}^{P}<<\gamma_{1}^{K}$), we can approximate condition 3) of Theorem 1 as $\frac{\alpha_{0}^{P}}{\beta_{0}^{P}}\left[S_{\text{tot}}\right]⪆\frac{n}{2(n+1)}\frac{k_{1}^{K}}{k_{0}^{K}k_{1}^{P}}\frac{\gamma_{1}^{P}\gamma_{2}^{P}}{\gamma_{0}^{K}\gamma_{1}^{K}}\approx 5\times 10^{-3}nM^{-1}$. (S1 Text, S1.3) The exact result, as also shown previously, is 4.88 × 10^−3^ *nM*^−1^. Here, $k_{i}^{K}=\frac{\beta_{i}^{K}+\gamma_{i}^{K}}{\alpha_{i}^{K}}$, $k_{i}^{P}=\frac{\beta_{i}^{P}+\gamma_{i}^{P}}{\alpha_{i}^{P}}$ are the Michaelis-Menten constants, which are inversely proportional to the rate constants for the production of enzyme-substrate intermediates from free enzymes and substrates (S1 Text, S1.1). This approximation is biologically meaningful and consistent with the mechanism described above for the two-site case. The right-hand side of this condition is smaller (making multistability less robust to substrate enzyme-sequestration) when *S*_1_ forms *KS*_1_ intermediates more readily than *PS*_1_ intermediates (i.e. small $k_{1}^{K}$, large $k_{1}^{P}$), when *S*_0_ has a low affinity for forming *KS*_0_ intermediates (i.e. large $k_{0}^{K}$), or when the competition for the phosphatase is higher and phosphatase is less readily made available from the intermediates than the kinase (i.e. low $\gamma_{1}^{P}$ and $\gamma_{2}^{P}$, high $\gamma_{0}^{K}$ and $\gamma_{1}^{K}$).

#### Substrate enzyme-sequestration effects are not necessarily limited to *S*_0_ and *S*_*n*_ or even to multi-site protein phosphorylation

**Fig 7:**
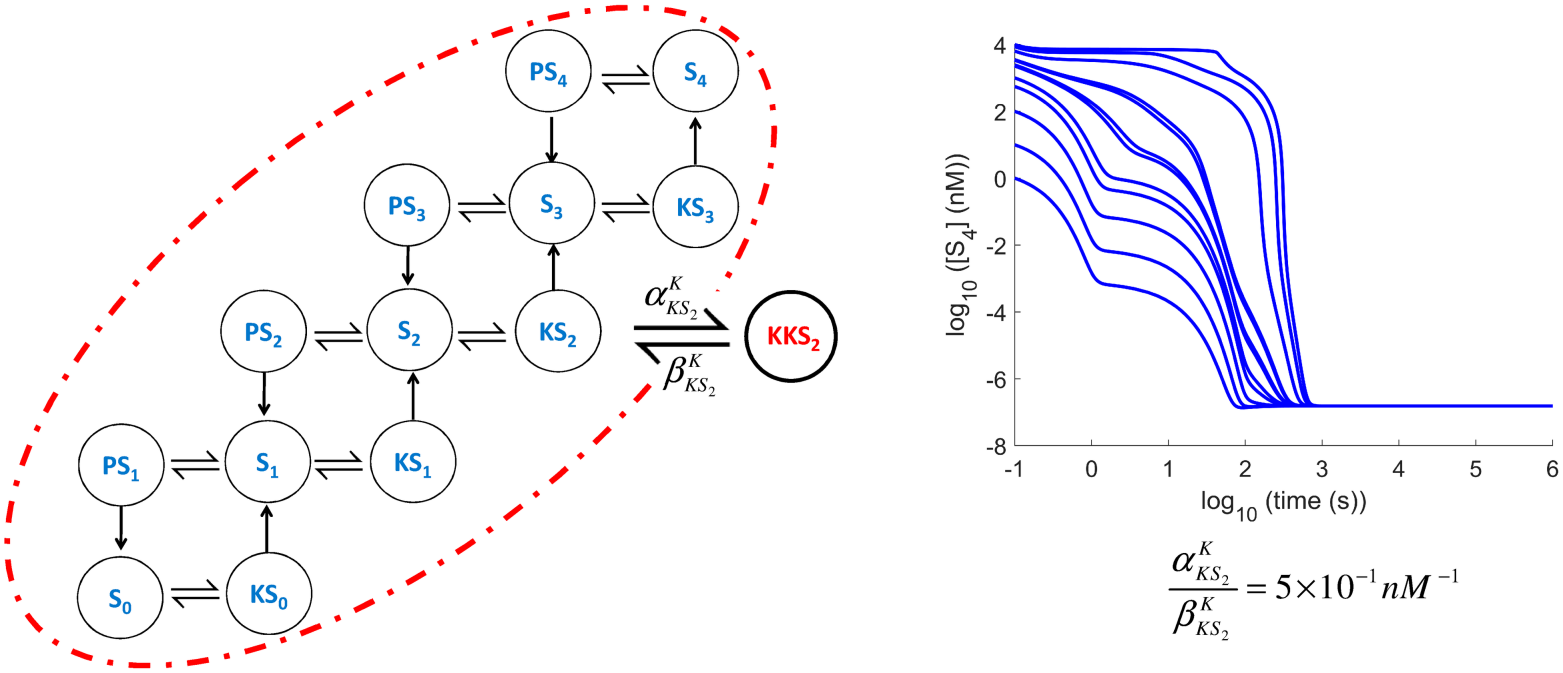
Sequestration of kinase by a kinase-substrate intermediate (*KKS*_2_ formation from *KS*_2_). This could also cause the system to lose its multistable behaviour, resulting to monostability.

Having identified that Substrate Enzyme-Sequestration introduces self-inhibition which disrupts the mechanism that caused multistability, one can see that other inactive complexes might also limit the extent of multistability. For example, if the intermediate complex *KS*_2_ is allowed to form an inactive complex *KKS*_2_ by using an allosteric secondary site perhaps, it is essentially competing with *S*2 for its own production. This effectively reduces the coupling provided via *KS*_2_. A small sequestration strength (5 × 10^−1^ *nM*^−1^ in Fig 7) results in monostability. Note though that not all inactive complexes would have this effect on the system. For example, if *KS*_2_ is allowed to bind with phosphatase *P* to form *PKS*_2_, then this does not have the same impact on the coupling via *KS*_2_. Indeed, in simulations, such an inactive complex formation even with sequestration strengths of the order of 1 × 10^3^ (i.e. 2000 times stronger affinity than before) did not affect the tristability of the original system.

**Fig 8:**
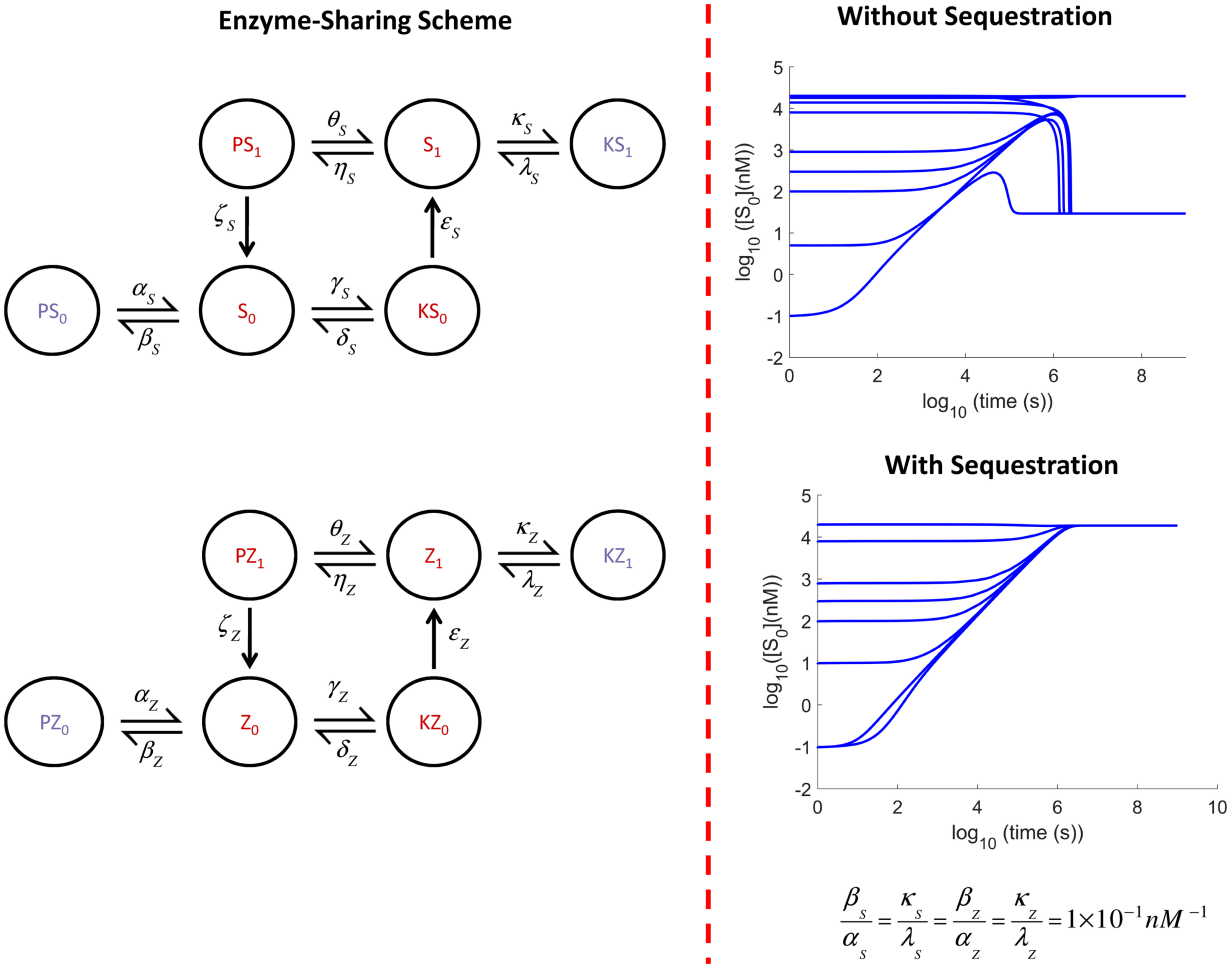
Effect of substrate enzyme-sequestration in a different mechanism. Substrate Enzyme-Sequestration can limit bistability in a different enzyme-sharing mechanism as well, where two substrates with one phosphosite compete for the same kinase and phosphatase.

The principle of enzyme competition when substrate is in excess is also prevalent in other enzyme-sharing schemes, for example where two substrates compete for the same kinase and phosphatase. To investigate this we took a one-site substrate model, which has been shown in the literature to exhibit bistability [7]. Following the conditions derived in that paper, we were indeed able to create bistability, which was then turned to monostability on the addition of Substrate Enzyme-Sequestration of strength 1 × 10^−1^ *nM*^−1^. This is illustrated in Fig 8. This can be explained along the same lines as before. For example, *S*_0_ can be thought of as an inhibitor of *Z*_1_ through competition with *Z*_0_ for the kinase, while *Z*_1_ is an inhibitor of *S*_0_ through competition with *S*_1_ for the phosphatase. The same applies to the pair *Z*_0_ and *S*_1_ as well. When *S*_0_, for example, is allowed to compete for the phosphatase in order to form the inactive complex *PS*_0_, it is self-inhibiting, gradually weakening the feedback loop with *Z*_1_.

#### Generalisation to arbitrary processivity and sequentiality

Since the new sequestration species added to the system do not interfere with its internal structure, we can in the same way extend the general framework of Thomson and Gunawardena [18], with arbitrary processivity and sequentiality (i.e. multiple phosphorylations or dephosphorylations can happen per reaction and in any order). The main conclusion, that increasing the strength of either kinase or phosphatase sequestration ultimately reduces the number of steady states to one, remains unchanged.

In this general framework the new three *ϕ* functions, are rational positive [18], allowing a rational expression in *u*, *R*(*u*), to be defined.

$$\begin{array}{c} 0=\left(u-w\right)\phi_{1}+\left[S_{\text{tot}}\right]\left(u\phi_{2}-w\phi_{3}\right)≕R\left(u\right) \end{array}$$

where $\left(w=\frac{\left[K_{\text{tot}}\right]}{\left[P_{\text{tot}}\right]}\right)$.

It was shown that *R*(*u*) can be expressed as $\frac{P(u)}{Q(u)}$, where *Q*(*u*) is an s-positive polynomial (sum of positive monomials). Therefore the steady states of the system can just be found by finding the roots of *P*(*u*), with *N* + 1 now lying between *n* + 1 and 2^n^ depending on the model [18].

$$\begin{array}{c} P\left(u\right)=a_{N+1}u^{N+1}+a_{N}u^{N}+\ldots +a_{1}u+a_{0} \end{array}$$ (5)

As before, the leading coefficient *a*_*N*+1_ is positive and the trailing coefficient *a*_0_ is negative, (S1 Text, S1.7). When phosphatase sequestration is added, the polynomial changes to

$$\begin{array}{c} P^{\prime }\left(u\right)=P\left(u\right)-\left[S_{\text{tot}}\right]w\frac{\alpha_{0}^{P}}{\beta_{0}^{P}}Q\left(u\right) \end{array}$$ (6)

Since more than one coefficient is changed it not possible to use the Pratt tableau directly as before. However, since *Q*(*u*) is s-positive, and so can’t itself have any positive real roots by the Descartes’ rule of signs, and its degree is less than that of *P*(*u*), it must be the case that *P*′(*u*) will have precisely one positive real root for sufficiently large $\frac{\alpha_{0}^{P}}{\beta_{0}^{P}}$ (S1 Text, S1.7).

The same argument applies to kinase sequestration, by relabelling the fully phosphorylated substrate as *S*_0_ and writing the polynomials in terms of *u*^−1^ = [*P*]/[*K*] instead.

### Substrate enzyme-sequestration in the stochastic domain

#### The behaviour of substrate enzyme-sequestration when the molecule numbers are small require a different analysis

So far we have proved that increasing the strength of Substrate Enzyme-Sequestration in multi-site phosphorylation systems leads to the monotonic decrease of whatever multistability would be possible if the inactive complex formation (*PS*_0_ and *KS*_*n*_) was not considered, no matter what the kinetic parameters. Ultimately, this monotonic decrease leads to one steady state. However, this analysis was done in the deterministic domain, which is technically only valid when the molecule numbers are infinite. Therefore, to obtain a full understanding of the effect of the studied Substrate Enzyme-Sequestration is essential that an accompanied analysis is done for the case when this assumption is not valid.

**Fig 9:**
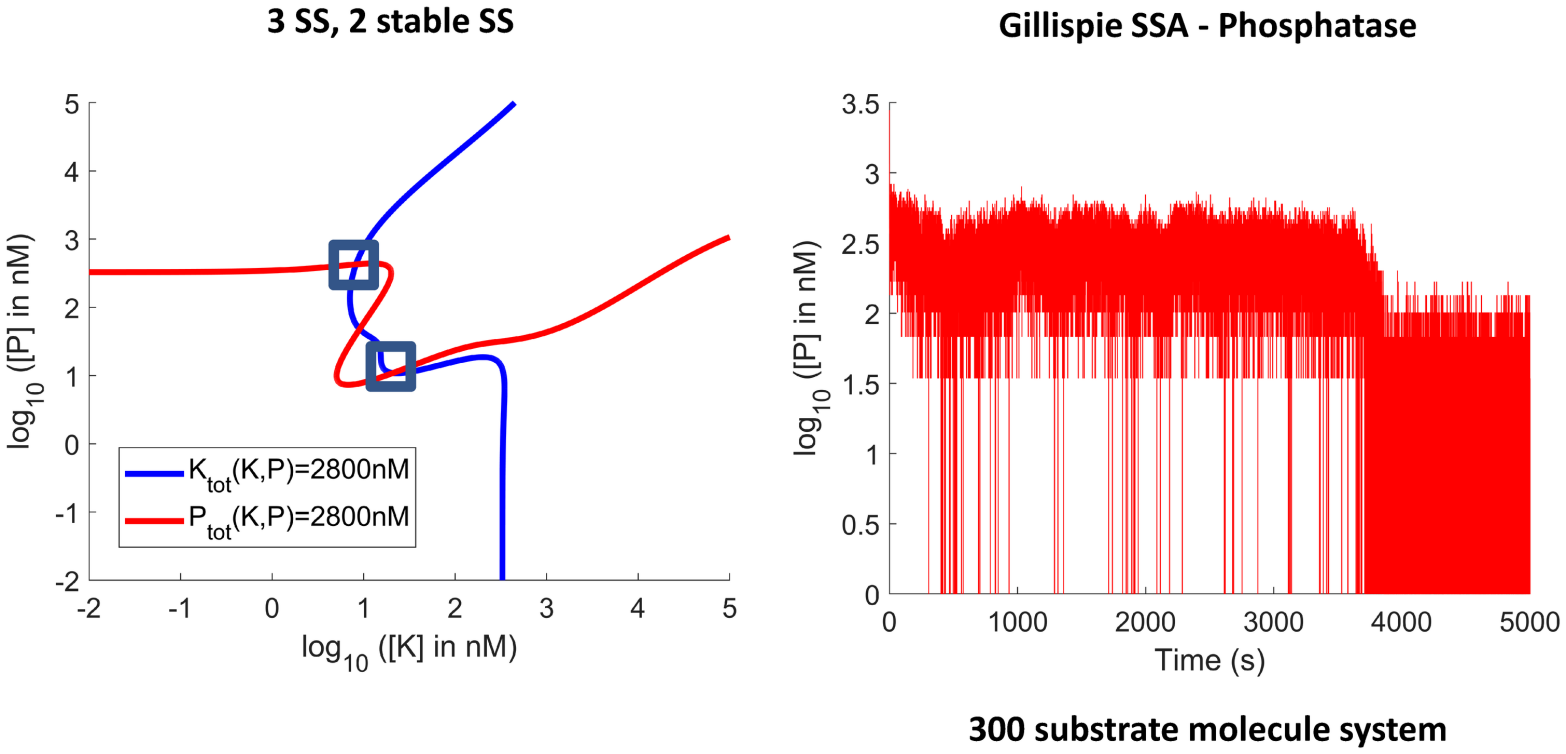
Bistability in the stochastic domain. The original tristable system is simulated in a system of 300 substrate molecules (with sequestration strengths of $\frac{\alpha_{n}^{K}}{\beta_{n}^{K}}=1\times 10^{-3}$ *nM*^−1^ and $\frac{\alpha_{0}^{P}}{\beta_{0}^{P}}=1\times 10^{-3}$ *nM*^−1^), corresponding to the original system in a volume of 4.98 × 10^−17^ L. Bistability in the deterministic domain (left) manifests itself as bimodality in the stochastic domain (right). The simulation was done using the Gillispie Stochastic Simulation Algorithm (SSA).

When molecule numbers are large but finite, bistability of the differential equations manifests itself as bimodality of the stochastic system. The modes correspond to the stable steady states of the system, and the system undergoes fluctuations within, and random jumps between, the modes. To illustrate this we use the original tristable system presented in [18, 32] (S1 Text S1.11). Considering substrate-kinase and substrate-phosphatase sequestration, with respective strengths $\frac{\alpha_{n}^{K}}{\beta_{n}^{K}}=1\times 10^{-3}$ *nM*^−1^ and $\frac{\alpha_{0}^{P}}{\beta_{0}^{P}}=1\times 10^{-3}$ *nM*^−1^, bistability (three steady states, two stable) is obtained, as shown by the intersections of the contours for [*K*_tot_] and [*P*_tot_] in Fig 9 (left), in a similar manner to [18]. The result of the stochastic simulation with the same numerical parameters (including the ratios between enzymes and substrate) but with the parameters converted to units of molecules instead of units of concentration is also shown in Fig 9 (right). The system can be seen to jump between the modes.

#### The same strength of substrate enzyme-sequestration, sufficient for monostability, can lead to both monomodality and bimodality in the stochastic domain, depending on the timescales of the individual sequestration parameters

When molecule numbers are small, however, there may be little relationship between the continuous deterministic and discrete stochastic analyses [37, 38]. A well-studied example is the genetic toggle switch, which in the absence of cooperativity it is predicted to have only one stable steady state [39], whereas experimental results and exact stochastic simulations have shown that the system exhibits bimodality instead [37, 40, 41]. This is usually referred to as ‘noise-induced’ bimodality. Understanding this bimodality is hard, yet even harder is the prediction of when this would take place [41]. Therefore, as our results demonstrate that Substrate Enzyme-Sequestration will ultimately lead to one steady state, it is imperative to check whether this mechanism has the same effect in the stochastic domain.

**Fig 10:**
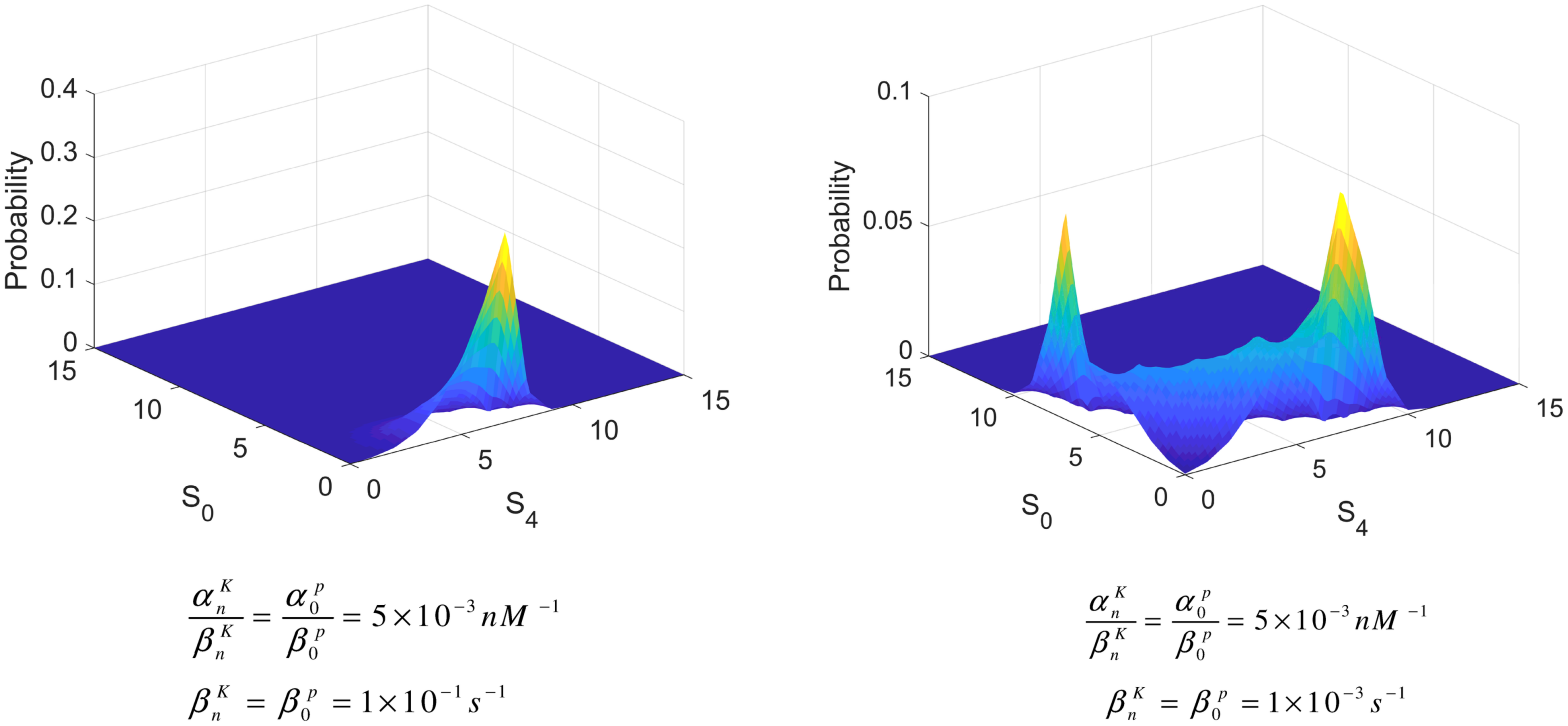
Timescale importance in stochastic analysis. In the stochastic domain, the same strength of sequestration can produce both monomodal (left), and bimodal (right) behaviour, depending on the individual timescales of the sequestration parameters. The ratio, unlike in the deterministic case, is not sufficient to deduce behaviour.

In order to investigate the effect of enzyme sequestration by the substrate in this regime we considered a 15-substrate molecule single phosphosite system, using the same parameters, wherever applicable, as in the original tristable system (in a volume of 2.49 × 10^−18^ L). Four kinase and four phosphatase molecules (thus having the same substrate/enzyme ratios as before) were selected. To examine whether the predicted monostability is obtained, we use a strength of sequestration found in earlier sections to be sufficient for monostability ($\frac{\alpha_{n}^{K}}{\beta_{n}^{K}}=\frac{\alpha_{0}^{P}}{\beta_{0}^{P}}=5\times 10^{-3}$ *nM*^−1^). For the same sequestration strength, two different behaviours emerged, depending on the timescale of the kinetic parameters used. This is different to the deterministic case, where the steady states are only dependent on the ratio. For $\beta_{n}^{K}=\beta_{0}^{P}=1\times 10^{-1}$ *s*^−1^, the result was a monomodal probability distribution, agreeing with the prediction from the deterministic analysis. However, when $\beta_{n}^{K}=\beta_{0}^{P}=1\times 10^{-3}$ *s*^−1^, for the same sequestration strength, a bimodal behaviour emerged, as illustrated in Fig 10. Note that, as shown in S1 Text, S1.11, $\beta_{i}^{P}$ and $\beta_{i}^{K}$ are of the order of 10^−3^ to 10^+0^.

This behaviour shows that the extra mode created is dependent on the time required to get out of that state i.e. the dwell time. This is because the free kinase is completely depleted, as it is trapped in intermediate complexes, leaving only free phosphatase around. In the absence of kinase and in the presence of just phosphatase, the substrate is kept in a mode where it is only unphosphorylated. This mechanism cannot be represented in the deterministic analysis, as the concentration of the enzymes never becomes exactly zero.

Nevertheless, this mechanism could serve a specific function when a system requires different behaviour when its size expands. In smaller sized systems, it could be beneficial that both unphosphorylated and phosphorylated substrate molecules are available, triggering different cascades of reactions. When the system becomes large however, one of the two mechanisms might be more beneficial.

Bimodality is induced when the kinase becomes extinct for a period of time, allowing the phosphatase without competition to completely dephosphorylate the available substrates. This mechanism does not appear to be dependent on the number of available phosphosites, therefore higher orders of multimodality due to this mechanism are not expected (and we were unable to find any). Nevertheless, it is possible to induce it with only one available phosphosite, impossible when the system is analysed deterministically, as we show in the next section where we formulate the intuition here into a mathematical framework. This provides a methodology to characterise parameter regimes where bimodality can be expected.

### A stochastic tool, separating the network inputs from the local outputs

In the previous section we noted that Substrate Enzyme-Sequestration and manipulation of dwell times is enough to create bimodality. In this section we formulate this intuition into a new framework which can allow bimodality be investigated in a methodological approach.

To do this we start from an accurate stochastic framework and reformulate it. Our intuition is that the stochastic effects result when a state with low outward transition rates, is visited often compared to the other states of the network. This suggests that our reformulation needs to separate the ‘network’ input from the ‘local’ output effects.

An accurate stochastic framework that does not depend on simulations is the discrete Chemical Master Equation, represented by a discrete state continuous time Markov process [42]. The microstate of the system (which is also described in literature, and below, simply as a ‘state’) involving *n* species is defined as $x\left(t\right)=\left\{x_{1}\left(t\right),x_{2}\left(t\right),\ldots ,x_{n}\left(t\right)\right\}\epsilon N^{n}$. A microstate is therefore describing a possible combination of the different population numbers of each molecular species in the system. The discrete Chemical Master Equation can be written in matrix vector form, as Eq 7, where ***A*** is built from the the positive transition rates, or *propensities*, from one state to another. Note that matrix ***A*** is a zero column sum (ZCS) square matrix, as the sum of the probabilities can not change (and must always equal 1).

$$\begin{array}{c} \frac{dP(t)}{dt}=\text{AP}\left(t\right) \end{array}$$ (7)

Thus, in order to find the stationary probability distribution **P**_**s**_ of an irreducible process in a finite state-space, applicable to our Substrate Enzyme-Sequestration problem, one can just solve Eq 8 by finding the null space of **A** [43] and then normalising so that the sum of probabilities of the states adds up to one. This is equivalent to finding the steady state of the system in the stochastic domain.

$$\begin{array}{c} \text{AP}(t)=0 \end{array}$$ (8)

**Fig 11:**
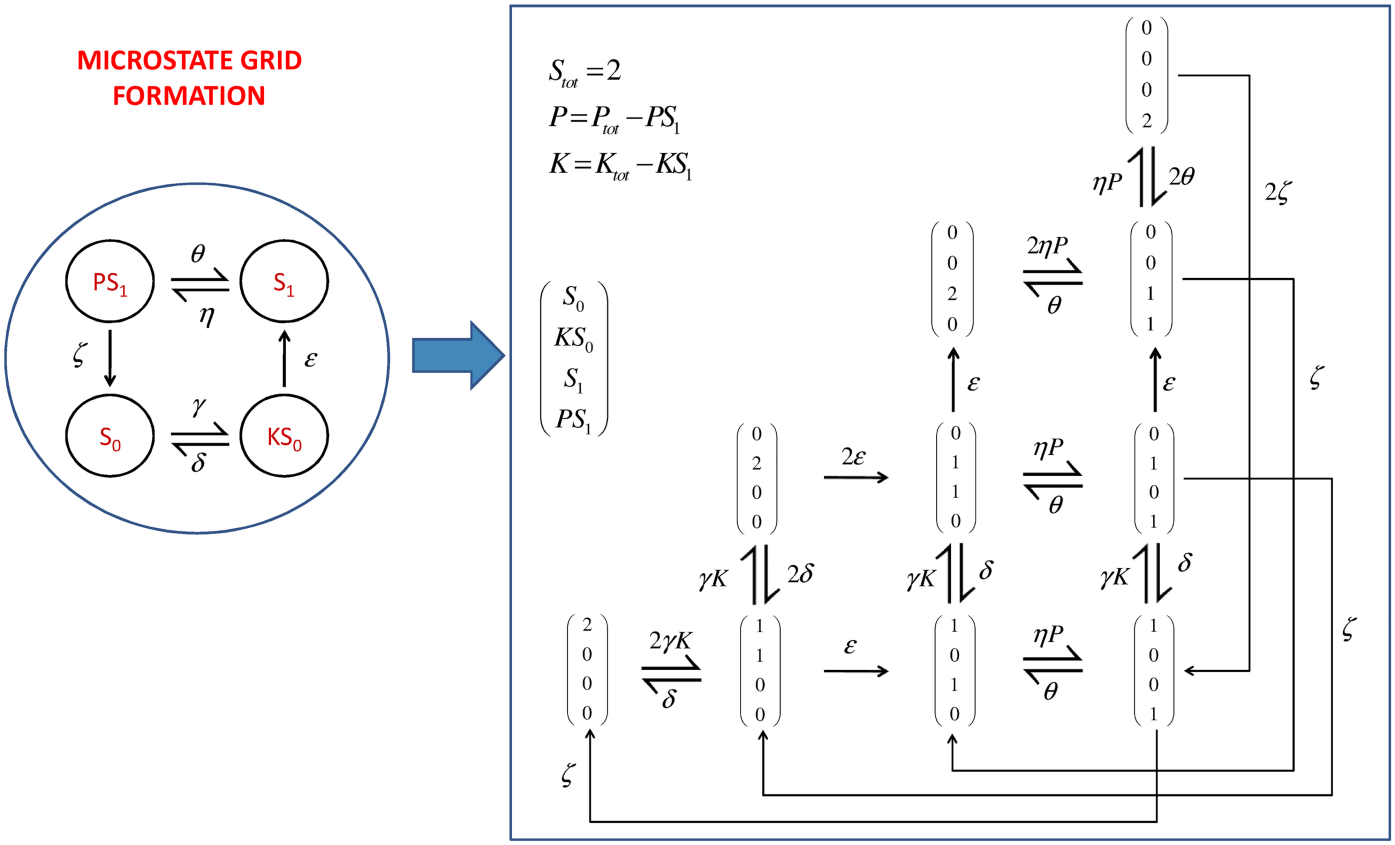
Reaction scheme to grid of microstates conversion. This illustrative example with just two substrate molecules and one available phosphorylation site shows how a reaction scheme is converted to a grid of microstates. This example assumes excess enzyme, therefore the microstates include all possible combinations, as there is no extra constraint.

The analysis of a stochastic system using the Chemical Master Equation framework requires firstly the conversion of the reaction scheme into a microstate grid. Fig 11 shows how this is done in the illustrative example of just two substrate molecules for a system with a single phosphosite. This example assumes excess enzyme, therefore the microstates include all possible combinations, as there is no extra constraint. When substrate is in excess (e.g. there is only one kinase molecule and two substrate molecules), the microstates representing enzyme complexes (e.g. *KS*_0_) greater than the total number of the corresponding enzyme (e.g. *K*) have to be deleted from the grid, as it is now not possible to have two *KS*_0_ molecules.

Our formulation extends a Proposition made by Karim et al [44]. The theoretical development of this tool, along with its associated theorems, can be found in S1 Text, S1.8. Matrix **A** contains all the information required to create the discrete state continuous time Markov process ruling the system. As we assume that the Markov chain is strongly connected, we can turn the calculation of the stationary probability distribution from a calculation of the null space of **A** to the solution of a linear system of equations.

This can done by solving $A_{j,j}^{D}q=-A_{j}^{j}$ where $A_{j,j}^{D}$ is the sub-matrix formed after deleting the *j*^*th*^ row and *j*^*th*^ column from matrix ***A*** and $A_{j}^{j}$ is the the *j*^*th*^ column of matrix ****A**** with element *j* deleted. *q* is a column vector of size (*n* − 1) and $P_{s}^{i}$ is the stationary probability of microstate *i*, *q* = [*q*_1_, *q*_2_, *q*_3_, …, *q*_*j*−1_, *q*_*j*+1_, *q*_*j*+2_, …, *q*_*n*_]^*T*^, $q_{k}=[\frac{P_{s}^{k}}{P_{s}^{j}}]$.

**Fig 12:**
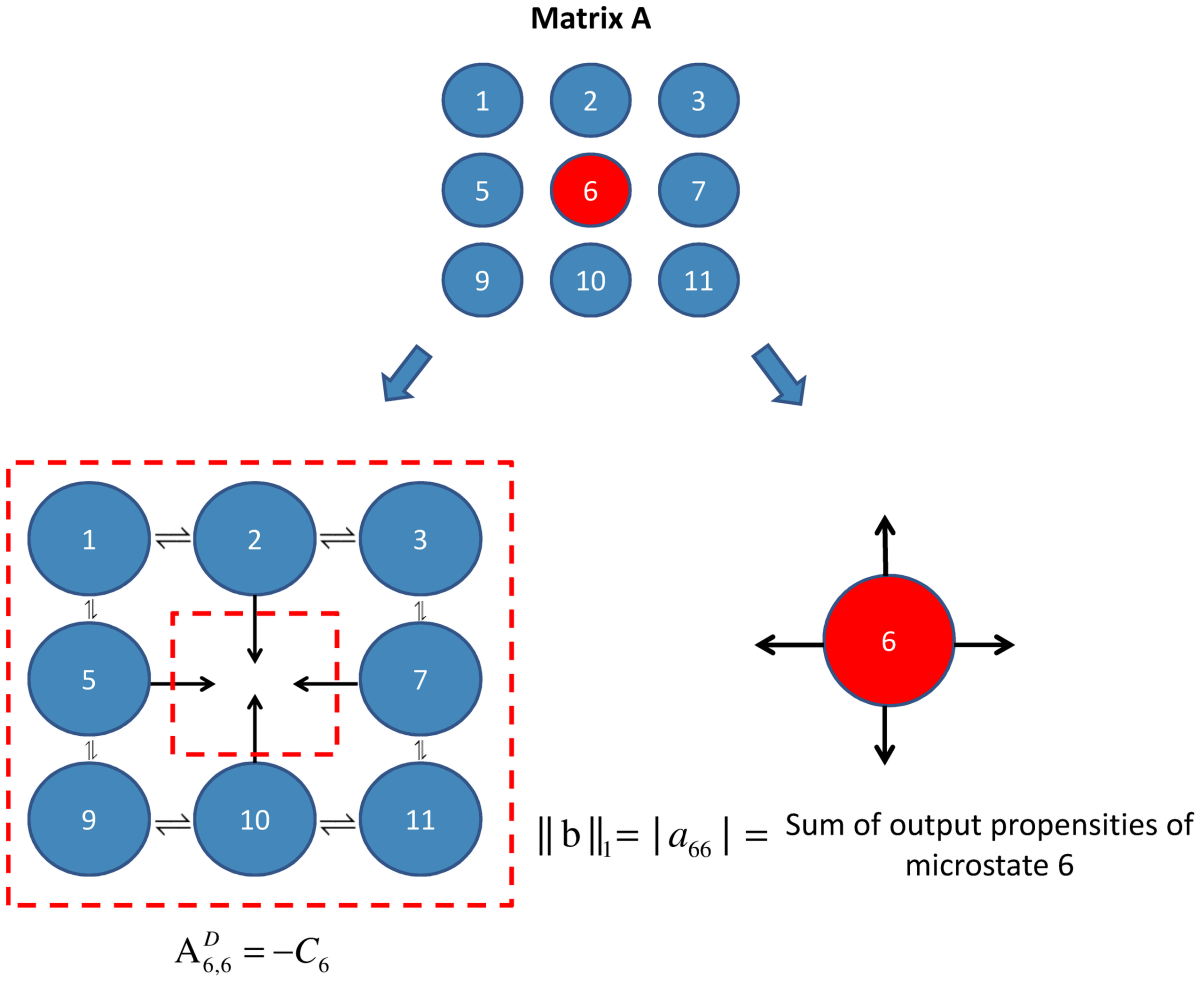
The source/sink analogy of the Chemical Master Equation’s formulation. The formulation of the Chemical Master Equation allows the separate investigation of the input effects of the rest of the network on the microstate under investigation from its local output propensities (a source/sink analogy).

This formulation separates, as initially aimed, the output propensities of a particular microstate from the rest of the parameters of the system, as graphically illustrated by Fig 12. This means that we can now bound the stationary probability of a particular microstate $P_{s}^{j}$ using spectral properties of $A_{j,j}^{D}$ and the magnitude of *a*_*jj*_ (the latter is simply the sum of the output propensities of microstate *j*). Let ${C_{j}=-A}_{j,j}^{D}$ and $b=A_{j}^{j}$ Then, defining λ_*i*_(***C***_***j***_)and λ_*min*_(***C***_***j***_) to be the *i*^*th*^ and the minimum eigenvalue of matrix ***C***_***j***_ respectively and knowing that matrix ***C***_***j***_ is an *m* × *m* matrix (where *m* = *n* − 1), $P_{s}^{j}\geq \frac{\lambda_{min}(C_{j})}{\lambda_{min}(C_{j})+m\left|a_{jj}\right|}$. Moreover, defining *σ*_*max*_(***C***_***j***_) to be the maximum singular value of matrix ***C***_***j***_, $P_{s}^{j}\leq \frac{\sigma_{max}(C_{j})}{\sigma_{max}(C_{j})+∥b{∥}_{2}}$.

The aforementioned results were obtained after proving that Matrix ${C_{j}}^{T}=-{\left(A_{j,j}^{D}\right)}^{T}$ is a weakly chained diagonally dominant (WCDD) M-matrix [45]. Intuitively, this means that the negated transpose of the matrix can be represented by a Markov chain, where there is a path from every microstate to reach at least one flux ‘hanging’ out of the grid, which is exactly what we observe in Fig 12.

An added benefit of this formulation lies in the fact that accurate algorithms can be developed for this class of matrices [46] in computing the singular values [47–49], the smallest eigenvalue [50] and the inverse [51]. The accuracy of these algorithms is independent of any condition number.

Observing the lower bound of the individual microstate probability, $\left(\frac{\lambda_{min}(C_{j}^{T})}{\lambda_{min}(C_{j}^{T})+m\left|a_{jj}\right|}\right)$, we can use the minimum eigenvalue of $C_{j}^{T}$ (for which an accurate computational algorithm is presented in [50]) as a means to capture the information about the input effect on the microstate from the entire grid, whereas |*a*_*jj*_|, the sum of the microstate’s output propensities, provides a measure of the dwell time spent in a microstate. *m* is a constant, therefore we use the ratio of $\frac{\lambda_{min}(C_{j}^{T})}{|a_{jj}|}$ to investigate the effect of the different parameters on the formation of the stationary probability distribution.

The sum of these ratios $\frac{\lambda_{min}(C_{j}^{T})}{|a_{jj}|}$, to be referred from now on as characterisation ratios, of the microstates corresponding to a particular substrate state can provide a fast and relatively accurate measure of how the stationary probability distribution for the substrate states varies with different macroscopic parameters (reaction rates).

### Bimodality is feasible even when only one phosphosite is available

**Fig 13:**
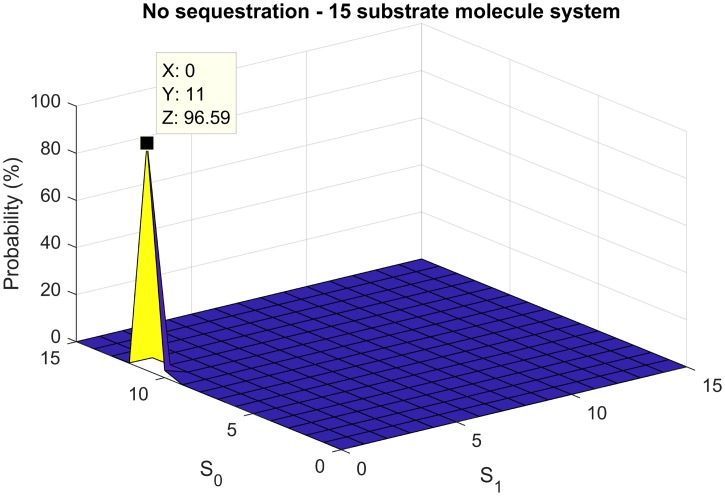
Monomodality of single phosphosite system with no sequestation. No sequestration leads to monomodality for a 15-substrate molecule single phosphosite system, with four kinase and four phosphatase molecules. This is consistent the monostability expected using the deterministic analysis.

The same 15-substrate molecule single phosphosite system was investigated as before (in a volume of 2.49 × 10^−18^ L), yet with only one phosphosite. Four kinase and four phosphatase molecules were again selected. This provides us, as expected from the deterministic analysis, only one mode at (*S*_0_, *S*_1_) = (11, 0). This is illustrated in Fig 13.

The tool developed allows the investigation to take place without considering all the substrate states (*S*_0_, *S*_1_) of the system. Instead, we can initially focus in just some of them. As the original mode (*S*_0_, *S*_1_) = (11, 0) is found at the boundary *S*_1_ = 0, we include the boundary states (*S*_0_, *S*_1_) = (6, 0) − (11, 0), in our analysis. As we aim for bimodality, we also include their reciprocal states on the other boundary *S*_0_ = 0, (*S*_0_, *S*_1_) = (0, 6) − (0, 11). Finally, we also include some states in between to establish that they do not become more dominant than the ones on the boundaries, e.g. (*S*_0_, *S*_1_) = (4, 3) or (3, 4).

**Fig 14:**
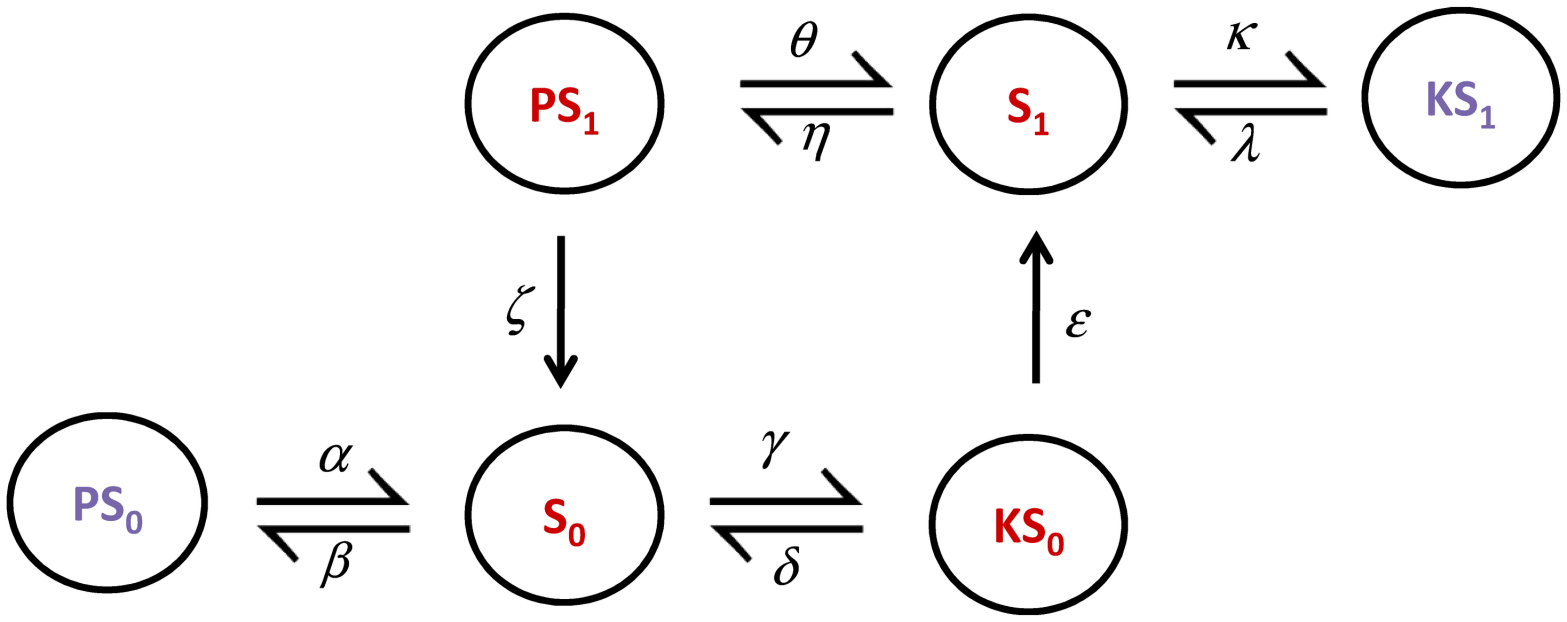
Extension of the one phosphosite system. The one phosphosite reaction scheme is extended to include the inactive complexes *PS*_0_ and *KS*_1_.

The first step is to set all the parameters of the reaction scheme, as shown in S1 Text, S1.11, letting only the sequestration parameter under investigation. This is *α*, as shown in the reaction scheme of Fig 14. Note that we vary this parameter (which captures the dwell time of the extra mode) instead of the ratio of sequestration strength, following the observation of Fig 10. The non-sequestration parameters are the same as the ones in the multisite protein phosphorylation system by Thomson and Gunawardena [18, 32].

**Fig 15:**
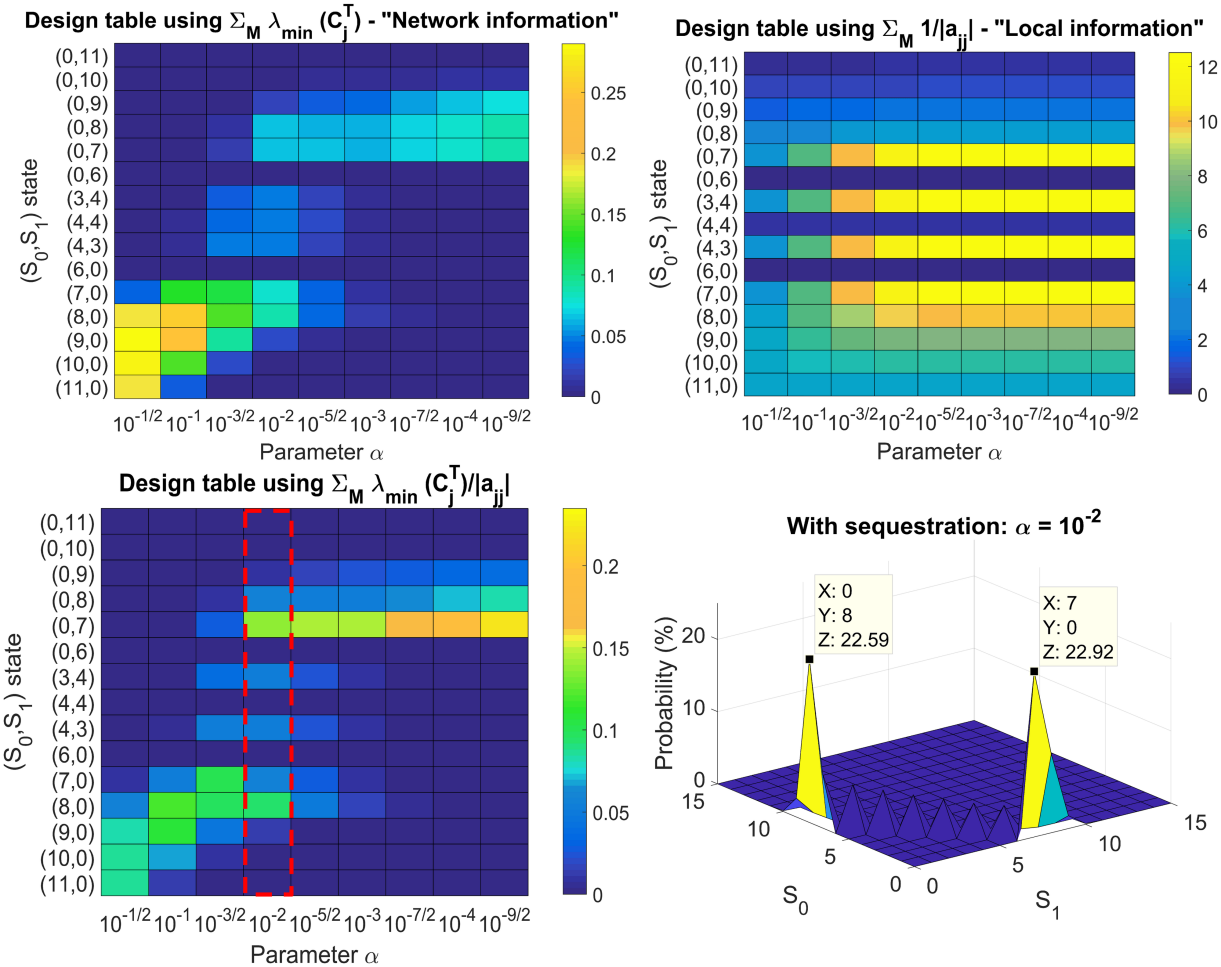
Design table using the characterisation ratios. The sum of the characterisation ratios corresponding to the microstates of different substrate states under investigation (bottom left) can be used to investigate the parameter regime of *α* for bimodality to occur. The top left and top right graphs illustrate the network input and local output effects, allowing for greater insights behind the creation of the two modes. The main driver making (*S*_0_, *S*_1_) = (8, 0) a mode is the effect of the inputs on the network-level, whereas the main driver making (*S*_0_, *S*_1_) = (0, 7) a mode is the low local output propensities of its corresponding microstates.

The next step is to create a design table (bottom left of Fig 15) using the sum the characterisation ratios $\frac{\lambda_{min}(C_{j}^{T})}{|a_{jj}|}$ for the *M* microstates corresponding to each substrate state as we vary *α*. The design table allows us to estimate the region of values of *α* that can allow bimodality. The result is shown in Fig 15, where it is found that at *α* = 10^−2^, bimodality can be obtained, creating modes at (*S*_0_, *S*_1_) = (8, 0), (0, 7) (bottom right). As mentioned before, the numerator of the characterisation ratio, which is the minimum eigenvalue of the developed WCDD M-matrix, provides a network input metric, whereas the denominator, |*a*_*jj*_| represents the local output propensities of the particular microstate. The top left and top right design tables illustrate the sum of the numerators and the sum of the inverse of the denominators of the characterisation ratios respectively. From these we can see that the main driver making (*S*_0_, *S*_1_) = (8, 0) a mode is the effect of the inputs on the network-level, whereas the main driver making (*S*_0_, *S*_1_) = (0, 7) a mode is the low local output propensities of its corresponding microstates.

Finally, we also verified that the bimodality of the system is robust to fluctuations in enzyme and substrate molecule numbers due to transcription, translation and decay, by extending our model to explicitly include those reactions as well. Indeed, the stochastic system continued to strongly exhibit a bimodal behaviour, yet with the fluctuations leading to less sharp modes, as expected. The extended model, together with the associated simulations and results can be found in S1 Text, S1.10.

### Conclusion

In this paper we first identified the effect of enzyme docking, and the Substrate Enzyme-Sequestration it implies, in the presence of excess substrate and in the regime of large molecule numbers, proving that increasing the strength of sequestration the extent of multistability is limited and ultimately reduced down to one steady state. Secondly, we explored the mechanism’s effect in the presence of small molecule numbers. For the latter, the analysis was naturally placed in the stochastic domain. For that, we note that the sequestration strength, represented as a ratio, cannot provide acccurate predictions by itself of the behaviour of the system. We found that the individual dwell times as compared to the spectral properties of the rest of the network need to be considered to identify the behaviour of sequestration. This observation was formalised in a mathematical framework, allowing for a methodology in identifying when bimodality is feasible in the small numbers regime, even when bistability is even deemed as impossible using deterministic analysis.

## Supporting information

S1 Text S1 includes the proofs and the derivations of the results presented, the algorithms developed as well as the parameters used in the simulations. (PDF)
