# Supplementary material for: Enzyme sequestration by the substrate: An analysis in the deterministic and stochastic domains
Source: PLoS Comput Biol. 2018 May 17;14(5):e1006107. doi: 10.1371/journal.pcbi.1006107 (PMC5976211; doi:10.1371/journal.pcbi.1006107)
Supplement: S1 Text — (PDF) [file pcbi.1006107.s001.pdf]

Enzyme Sequestration by the Substrate: An Analysis in the Deterministic and Stochastic Domains

Andreas Petrides[1], Glenn Vinnicombe[1*],

1 Department of Engineering, University of Cambridge, United Kingdom

* gv@eng.cam.ac.uk

Supporting Information S1

S1.1 Deterministic Framework

In this section we summarise the deterministic framework used for sequential distributive multisite protein phosphorylation [1] and how this changes when Enzyme Sequestration by the Substrate is included. In sequential (de)phosphorylation, phosphosites are (de)phosphorylated in a strict order where (de)phosphorylation of one site depends on the phosphorylation state of another. This is opposite to a random (de)phosphorylation scheme. Distributive (de)phosphorylation occurs when the enzyme dissociates after each (de)phosphorylation. This is opposite to processive (de)phosphorylation where multiple (de)phosphorylations might take place before the enzyme dissociates from the substrate [2]. A sequential distributive system with four available phosphosites is presented in Fig. A.

Such a system is built from of two kinds of reaction: firstly, a kinase molecule K can attach to a substrate molecule with i phosphorylated phosphosites, S_i. The new complex formed, KS_i, can then either decompose back to K and S_i or phosphorylation can proceed, leading to the products K and S_{i+1}.

$$K + S_i \underset{\beta_i^K}{\overset{\alpha_i^K}{\rightleftharpoons}} KS_i \overset{\gamma_i^K}{\rightarrow} S_{i+1} + K$$

In addition, a phosphatase molecule P can attach to a substrate molecule with $i+1$ phosphorylated phosphosites, S_{i+1} with the new complex formed PS_{i+1} either decomposing back to P and S_{i+1} or lead to a dephosphorylation reaction with products P and S_i.

$$P + S_{i+1} \underset{\beta_{i+1}^P}{\overset{\alpha_{i+1}^P}{\rightleftharpoons}} PS_{i+1} \overset{\gamma_{i+1}^P}{\rightarrow} S_i + P$$

Under the assumption of excess substrate, i.e. that the total concentration of substrate $[S_{\text{tot}}] \gg [K_{\text{tot}}]$ and $[S_{\text{tot}}] \gg [P_{\text{tot}}]$, Thomson and Gunawardena [1] showed that the steady states of a phosphorylation system can be determined by the roots of the following polynomial (where $u = [K]/[P]$ and $w = \frac{[K_{\text{tot}}]}{[P_{\text{tot}}]}$),

$$P(u) = a_{n+1}u^{n+1} + a_n u^n + ... + a_1 u + a_0 \tag{S1}$$

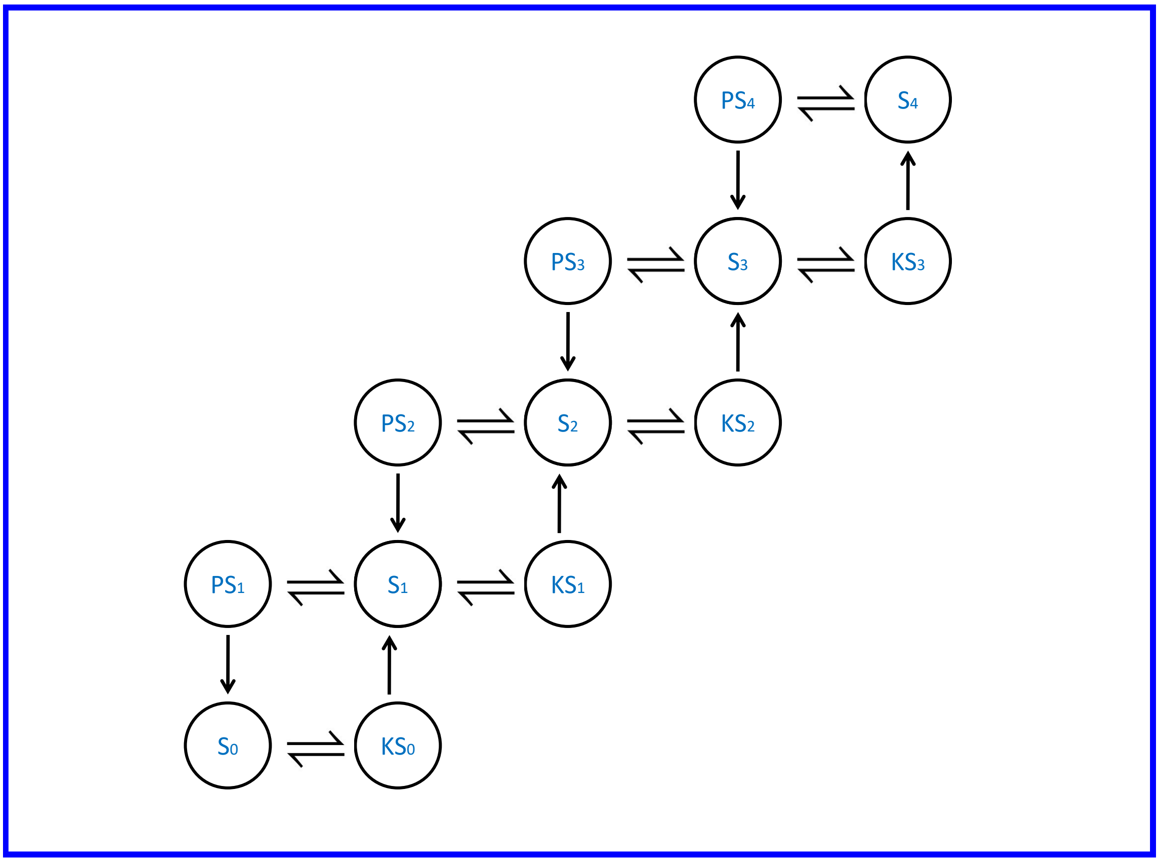

Figure A. A 4-site sequential distributive protein phosphorylation scheme

The expressions for the coefficients a_i are given in [1] as

$$\begin{aligned}
a_{n+1} &= \lambda_0\lambda_1...\lambda_{n-2}\lambda_{n-1}\\
a_0 &= -w\\
a_{i+1} &= \lambda_0\lambda_1...\lambda_{i-2}\lambda_{i-1}\left[(1-\lambda_i w) + [S_{\text{tot}}]\left(\frac{1}{k_i^K} - \frac{\lambda_i w}{k_{i+1}^P}\right)\right],\\
&\quad 0 \le i < n, \lambda_{-1} = 1
\end{aligned}$$

where

$$\lambda_i = \left(\frac{\gamma_i^K}{k_i^K}\right)\left(\frac{\gamma_{i+1}^P}{k_{i+1}^P}\right)^{-1},\ k_i^K = \frac{\beta_i^K + \gamma_i^K}{\alpha_i^K},\ k_i^P = \frac{\beta_i^P + \gamma_i^P}{\alpha_i^P} \tag{S2}$$

For completeness, these can be derived as follows:

Using mass kinetics, the steady state concentration of KS_i can be determined from the concentrations of the free kinase, K, and of the free substrate with i full phosphosites, S_i.

$$[KS_i] = \frac{[K][S_i]}{k_i^K},$$

Similarly for the phosphatase,

$$[PS_i] = \frac{[P][S_i]}{k_i^P},$$

At steady state, in each cycle the net flux into S_{i+1} must be equal to the net flux out of S_{i+1}. Consequently, taking into consideration each cycle in turn, one can express the concentration of any substrate state, $[S_{i+1}]$ as a function of the concentration of the substrate with no phosphorylated phosphosites, $[S_0]$, i.e.

$$[S_{i+1}] = [S_0]\lambda_0\lambda_1...\lambda_i\left(\frac{[K]}{[P]}\right),$$

Using the conservation of mass, we can write the total substrate concentration in terms of the individual species

$$[S_{\text{tot}}] = [S_0] + ... + [S_n] + [KS_0] + ... + [KS_{n-1}] + [PS_1] + ... + [PS_n] \tag{S3}$$

$$= [S_0]\left(\phi_1 + [K]\phi_2 + [P]\phi_3\right) \tag{S4}$$

where

$$\phi_1 = \sum_{i=0}^{n}\left(\prod_{j=0}^{i-1}\lambda_j\right)u^i$$

$$\phi_2 = \sum_{i=0}^{n-1}\frac{\left(\prod_{j=0}^{i-1}\lambda_j\right)u^i}{k_i^K}$$

$$\phi_3 = \sum_{i=1}^{n}\frac{\left(\prod_{j=0}^{i-1}\lambda_j\right)u^i}{k_i^P}$$

and u is the ratio of free kinase to free phosphatase ($u = \frac{[K]}{[P]}$).
The total concentrations of the kinase and phosphatase can be expressed in terms of the same functions:

$$[K_{\text{tot}}] = [K]\left(1 + [S_0]\phi_2\right) = [K]\left(1 + \frac{[S_{\text{tot}}]\phi_2}{\phi_1 + [K]\phi_2 + [P]\phi_3}\right) \tag{S5}$$

(using Eq. S4)

$$=: F_1\left([P],[K]\right) \tag{S6}$$

That is, the total concentration of the kinase is regarded as a function of free kinase and phosphatase concentrations. Similarly,

$$[P_{\text{tot}}] = [P]\left(1 + [S_0]\phi_3\right) = [P]\left(1 + \frac{[S_{\text{tot}}]\phi_3}{\phi_1 + [K]\phi_2 + [P]\phi_3}\right) \tag{S7}$$

$$=: F_2\left([P],[K]\right) \tag{S8}$$

A point $([P],[K])$ that satisfies $F_1\left([P],[K]\right) = [K_{\text{tot}}]$, $F_2\left([P],[K]\right) = [P_{\text{tot}}]$ is a possible steady state for the system and corresponds to an intersection of contours of F_1 and F_2 in a free phosphatase-free

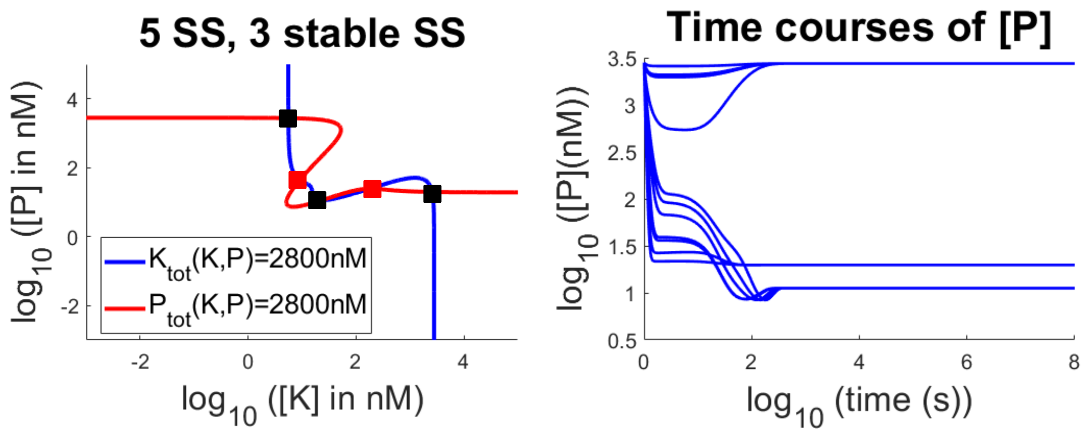

Figure B. Plots of total concentrations of kinase and phosphatase as functions of free kinase and phosphatase (left). Intersections correspond to steady states. The stable steady states are coloured in black, whereas the unstable steady states are coloured in red. The stable steady states are the steady states appearing in the time courses (right) when the system is simulated deterministically, starting from different initial conditions.

kinase map. This can be shown in Fig. B, where the stable steady states correspond to the steady states of the time courses when the system is deterministically simulated for the the original tristable system presented in [3]. Different steady states result according to the starting conditions.

As $\frac{\phi_1}{\phi_1+[K]\phi_2+[P]\phi_3} = \frac{[S_{\rm tot}]-[S_0][K]\phi_2-[S_0][P]\phi_3}{[S_{\rm tot}]}$, $[S_0][K]\phi_2 < [K_{\rm tot}]$ and $[S_0][P]\phi_3 < [P_{\rm tot}]$, in the regime of excess substrate (i.e. $[K_{\rm tot}] << [S_{\rm tot}]$ and $[P_{\rm tot}] << [S_{\rm tot}]$), the expressions of $[K_{\rm tot}]$ and $[P_{\rm tot}]$ can be approximated as follows:

$$[K_{tot}] = [K]\left(1 + [S_{tot}]\frac{\phi_2}{\phi_1}\right)$$

$$[P_{tot}] = [P]\left(1 + [S_{tot}]\frac{\phi_3}{\phi_1}\right)$$

Dividing the two expressions and rearranging, we obtain:

$$0 = (u-w)\phi_1 + [S_{tot}](u\phi_2 - w\phi_3) =: P(u)$$

where $(w = \frac{[K_{tot}]}{[P_{tot}]})$.

As ϕ_1, ϕ_2, and ϕ_3 are functions of u, the above expression can be rewritten as a univariate polynomial of u of order $n+1$.

$$P(u) = a_{n+1}u^{n+1} + a_n u^n + ... + a_1 u + a_0$$

$$a_{n+1} = \lambda_0\lambda_1...\lambda_{n-2}\lambda_{n-1}$$
$$a_0 = -w$$
$$a_{i+1} = \lambda_0\lambda_1...\lambda_{i-2}\lambda_{i-1}\left[(1-\lambda_i w) + [S_{tot}]\left(\frac{1}{k_i^K} - \frac{\lambda_i w}{k_{i+1}^P}\right)\right], 0 \leq i < n, \lambda_{-1} = 1$$

Taking the additional species into account, as in Fig. 2, ϕ_2 and ϕ_3 need to just be modified in terms of the summation indices to include all n substrate states in the mass conservation equations. Defining $k_n^K = \frac{\beta_n^K}{\alpha_n^K}$ and $k_0^P = \frac{\beta_0^P}{\alpha_0^P}$ for the new species, the ϕ functions become:

$$\hat{\phi}_2 = \sum_{i=0}^{n} \frac{\left(\prod_{j=0}^{i-1}\lambda_j\right)u^i}{k_i^K}$$
$$\hat{\phi}_3 = \sum_{i=0}^{n} \frac{\left(\prod_{j=0}^{i-1}\lambda_j\right)u^i}{k_i^P}$$

Updating accordingly the framework provided in the new polynomial, applicable for the regime, was found, as shown in Eq. S9. The new polynomial coefficients were calculated in terms of the old coefficients in order to allow for comparisons, providing an insight of the quantitative effect of Substrate Enzyme-Sequestration. Note that only the first and the last coefficients are changed.

$$P'(u) = a'_{n+1}u^{n+1} + a_n u^n + ... + a_1 u + a'_0 \tag{S9}$$

where

$$a'_{n+1} = a_{n+1}\left(1 + [S_{\text{tot}}]\frac{\alpha_n^K}{\beta_n^K}\right)$$
$$a'_0 = a_0\left(1 + [S_{\text{tot}}]\frac{\alpha_0^P}{\beta_0^P}\right)$$

S1.2 Theoretical development of the conditions on limiting the extent of multistability

Lemma S1.1. *If* $\frac{1}{1+\frac{\alpha_n^K}{\beta_n^K}[S_{tot}]} < \frac{2(n+1)a_{n+1}a_{n-1}}{na_n^2}$ *or* $\frac{1}{1+\frac{\alpha_0^P}{\beta_0^P}[S_{tot}]} < \frac{2(n+1)a_0a_2}{na_1^2}$ *then the number of real steady states is less than or equal to n-1.*

Proof. Suppose that $\frac{1}{1+\frac{\alpha_n^K}{\beta_n^K}[S_{\text{tot}}]} < \frac{2(n+1)a_{n+1}a_{n-1}}{na_n^2}$ and that all $n+1$ roots of Eq S9 are real.

Let x_i represent the ith root of Eq. S9. Then by the Vieta Formulae,

$$\left(\frac{a'_n}{a'_{n+1}}\right)^2 = \left(\sum_i x_i\right)^2 = \sum_i x_i^2 + 2\sum_{i<j} x_i x_j$$
$$\Rightarrow \sum_i x_i^2 = \left(\frac{a'_n}{a'_{n+1}}\right)^2 - 2\sum_{i<j} x_i x_j$$
$$\Rightarrow \sum_i x_i^2 = \left(\frac{a'_n}{a'_{n+1}}\right)^2 - 2\frac{a'_{n-1}}{a'_{n+1}}$$

Then by the Quadratic Mean - Arithmetic Mean and the Triangle Inequalities,

$$\sqrt{\frac{\sum_i x_i^2}{n+1}} \geq \frac{\sum_i |x_i|}{n+1} \geq \frac{|\sum_i x_i|}{n+1}$$
$$\Rightarrow \frac{n}{2}\sum_i x_i^2 \geq \sum_{i<j} x_i x_j$$

Using the obtained Vieta Formulae,
$\frac{n}{2}\left[\left(\frac{a'_n}{a'_{n+1}}\right)^2 - 2\frac{a'_{n-1}}{a'_{n+1}}\right] \geq \frac{a'_{n-1}}{a'_{n+1}}$
$\Rightarrow a_n^2 \geq 2a'_{n+1}a_{n-1}\left(\frac{n+1}{n}\right)$
Expressing it in terms of the old coefficients,
$a_n^2 \geq 2a_{n+1}a_{n-1}\left(1+[S_{\text{tot}}]\frac{\alpha_n^K}{\beta_n^K}\right)\left(\frac{n+1}{n}\right)$
$\Rightarrow \frac{1}{1+\frac{\alpha_n^K}{\beta_n^K}[S_{\text{tot}}]} \geq \frac{2(n+1)a_{n+1}a_{n-1}}{na_n^2}$ which is a contradiction. Therefore the roots x_i cannot all be real. Thus, due to the expectation of at least one conjugate pair of complex roots, the maximum possible number of positive real roots is less than or equal to $n-1$.
Similarly, $\frac{1}{1+\frac{\alpha_0^P}{\beta_0^P}[S_{\text{tot}}]} < \frac{2(n+1)a_0a_2}{na_1^2}$ is proved by considering the real roots of $P(v)$, $v=\frac{1}{u}$,

$$P(v) = a'_{n+1} + a_n v + ... + a_1 v^n + a'_0 v^{n+1}.$$

Note that instead of using the Vieta formulae, one could also use a general result proved using differential calculus in Section 4.3 of Hardy's 'Inequalities' book [4].

□

Theorem S1.2. *If any of the following conditions are satisfied, then the number of positive steady states will be no more than $n-1$ if n is even, or $n-2$ if n is odd:*

1. $a_{n-1} \leq 0$ *and* $a_2 \geq 0$
2. $a_{n-1} > 0$ *and* $\frac{\alpha_n^K}{\beta_n^K}[S_{tot}] > \frac{na_n^2 - 2(n+1)a_{n+1}a_{n-1}}{2(n+1)a_{n+1}a_{n-1}}$ *or*
3. $a_2 < 0$ *and* $\frac{\alpha_0^P}{\beta_0^P}[S_{tot}] > \frac{na_1^2 - 2(n+1)a_0a_2}{2(n+1)a_0a_2}$

Proof. From Eq. S9, $a_{n+1} > 0$ and $a_0 < 0$.
For even n, there is a maximum of an even number $(n+2)$ of coefficients, therefore a maximum of $n+1$ sign changes is possible. As $a_0 < 0$, then if $a_2 \geq 0$ then the last three coefficients, a_0, a_1 and a_2, can exhibit a maximum of one sign change. Knowing that there are a maximum of n coefficients from a_{n+1} to a_2, n being even and $a_{n+1} > 0$, $a_2 \geq 0$, then they can exhibit a maximum of $n-2$ sign changes. Therefore the total number of sign changes that can be exhibited is equal to $n-1$. From the Descartes' rule of signs the result follows immediately. Similarly for $a_{n-1} \leq 0$.

For odd n, there is a maximum of an odd number $(n+2)$ of coefficients, and as $a_{n+1} > 0$ and $a_0 < 0$, a maximum of n sign changes is possible. As $a_0 < 0$, then if $a_2 \geq 0$ then the last three coefficients, a_0, a_1 and a_2, can exhibit a maximum of one sign change. Similarly for the first three coefficients, $a_{n+1} > 0$, a_n and a_{n-1}, for $a_{n-1} \leq 0$. Knowing that there are a maximum of $n-2$ coefficients from a_{n-1} to a_2, n being odd, and $a_{n-1} \leq 0$, $a_2 \geq 0$, then they can exhibit a maximum of $n-4$ sign changes. Therefore the total number of sign changes that can be exhibited is equal to $n-2$. From the Descartes' rule of signs the result follows immediately.

The last two parts of the Theorem are directly derived from Lemma S1.1. For odd n, as $a_{n+1} > 0$ and $a_0 < 0$, there can be a maximum of n sign changes. The next number of sign changes possible is $n-2$. Therefore, from the Descartes' rule of signs it is not possible to have $n-1$ positive roots, as the parity of positive real roots and number of sign changes must be the same. The maximum therefore number of positive roots drops to $n-2$.

□

S1.3 Representing the derived conditions for limitation of multistability with kinetic parameters

To obtain a better intuition of the conditions derived in the previous section, we expand one of them in its corresponding kinetic parameters. Then we try to approximate the expression until we obtain a meaningful simple form. The approximation is valid for the parameters of the model (as in Section S1.11) i.e for equal concentrations of kinase and phosphatase ($w = 1$), S_{tot} being large and $\gamma_0^K << \gamma_1^P$, $\gamma_2^P << \gamma_1^K$). The condition we use here is the one associated with the inactive complex PS_0. The condition for the limitation of multistability in that case is that $a_2 < 0$ and $\frac{\alpha_0^P}{\beta_0^P} > \frac{na_1^2 - 2(n+1)a_0a_2}{2(n+1)a_0a_2[S_{\text{tot}}]}$.

$$\frac{a_1^2}{a_0a_2} = \frac{\alpha_2^P\gamma_2^P(\beta_1^K+\gamma_1^K)(\alpha_1^P\gamma_1^P(\beta_0^K+\gamma_0^K+a_0^K[S_{tot}])-w\alpha_0^K\gamma_0^K(\beta_1^P+\gamma_1^P+a_1^P[S_{tot}]))^2}{w\alpha_0^K\alpha_1^P\gamma_0^K\gamma_1^P(\beta_0^K+\gamma_0^K)(\beta_1^K+\gamma_1^P)(-\alpha_2^P\gamma_2^P(\beta_1^K+\gamma_1^K+\alpha_1^K[S_{tot}])+\alpha_1^K\gamma_1^K w(\beta_2^P+\gamma_2^P+\alpha_2^P[S_{tot}])}$$

Using that S_{tot} is large,

$$\begin{aligned}\frac{a_1^2}{a_0a_2} &\approx \frac{\beta_1^K+\gamma_1^K}{(\beta_0^K+\gamma_0^K)(\beta_1^P+\gamma_1^P)}\frac{\gamma_2^P}{\gamma_0^K\gamma_1^P}\frac{\alpha_1^P\alpha_0^K}{\alpha_1^K}\frac{(\gamma_1^P-\gamma_0^K w)^2}{\gamma_1^K w-\gamma_2^P}\frac{[S_{tot}]}{w}\\ &= \frac{k_1^K}{k_0^K k_1^P}\frac{\gamma_2^P}{\gamma_0^K\gamma_1^P}\frac{(\gamma_1^P-\gamma_0^K w)^2}{\gamma_1^K w-\gamma_2^P}\frac{[S_{tot}]}{w}\end{aligned}$$

Using that $w = 1$ and $\gamma_0^K << \gamma_1^P$, $\gamma_2^P << \gamma_1^K$ (from Section S1.11),

$$\begin{aligned}\frac{na_1^2 - 2(n+1)a_0a_2}{2(n+1)a_0a_2[S_{tot}]} &= \frac{na_1^2}{2(n+1)a_0a_2[S_{tot}]} - \frac{1}{[S_{tot}]}\\ &\approx \frac{na_1^2}{2(n+1)a_0a_2[S_{tot}]}\\ &\approx \frac{n}{2(n+1)}\frac{k_1^K}{k_0^K k_1^P}\frac{\gamma_1^P\gamma_2^P}{\gamma_0^K\gamma_1^K} \approx 5\times10^{-3}nM^{-1}\end{aligned}$$

The exact result is $4.88 \times 10^{-3}nM^{-1}$.

S1.4 Results based on the Pratt's tableau

Given any real polynomial, $P(x) = \sum_{j=0}^{n} c_j x^j$, a family of upper bounds on the number of roots in $(0,\infty)$ can be obtained using a Pratt tableau [5]. This can be created as follows:
Start with

$c_{n+1-j,j} = c_j$, $0 \le j \le n$
$c_{i,0} = c_0$ for all i
and $c_{0,j} = 0$ for $j > n$
Then by successive additions,
$c_{i,j} = c_{i,j-1} + c_{i-1,j}$ for $i \ge 1$, $j \ge 1$, $i + j > n + 1$

Definition S1.3. [5] An allowed path is any path through the tableau of the $c_{i,j}$ going from any $c_{i,0}$ to any $c_{1,j}$ with $j \ge n$ by horizontal, vertical and diagonal steps.

Theorem S1.4. *[5] The number of changes of sign (ignoring 0's) along any allowed path through the tableau generated by the coefficients of P(x) is an upper bound on the number of roots of P(x) in the relevant domain $(0,\infty)$, including multiplicities.*

Fact S1.5. *In the Pratt's tableau of any real polynomial, $P(x) = \sum_{j=0}^{n} c_j x^j$, the coefficient of c_j in the summation required for any entry k in the column j of the tableau is equal to 1.*

Theorem S1.6. *Let row m be the first row in which all elements of columns 0 to $n-1$ of the Pratt tableau of any real polynomial $P(x) = \sum_{j=0}^{n} c_j x^j$ with $c_0 > 0$ and $c_n < 0$ become positive. Let y be the nth column of the tableau considering the first $(m-1)$ rows. Then if $\delta \geq \frac{-max(y)}{c_n}$, then the real polynomial $P(x) + c_n\delta$ has fewer than 2 roots in the $(0, \infty)$ domain.*

Proof. Using the Pratt's tableau, if $c_0 > 0$, all rows will eventually become positive due to the successive additions $c_{i,j} = c_{i,j-1} + c_{i-1,j}, i \geq 1, j \geq 1$. Let the mth row denote the row in which all elements of columns 0 to $n-1$ become positive. If there is no sign change change in the nth column up to the $(m-1)^{th}$ row, then the upper bound obtained by Theorem S1.4, is equal to one, as there will be no sign change across the mth row from $c_{m,0}$ to $c_{m,n-1}$ before having one sign change moving from $c_{m,n-1}$ to $c_{1,n}$. Creating an initial Pratt tableau of $P(x) = \sum_{j=0}^{n} c_j x^j$ with $c_0 > 0$ and $c_n < 0$ and letting y be the tableau's n^{th} column up to the $(m-1)^{th}$ row, then
From Fact S1.5,

$max(n^{th}column) = max(y) = c_n + V$, where $V \in \mathbb{R}$

For the nth column up to the $(m-1)^{th}$ row of the Pratt tableau of the real polynomial $P(x) + c_n\delta$ to always be negative or zero,

$c_n(1 + \delta) + V \leq 0,$
$c_n(1 + \delta) \leq -V$
$c_n(1 + \delta) \leq c_n - max(y)$
$\Rightarrow \delta \geq \frac{-max(y)}{c_n}$, as $c_n < 0$.

□

Corollary S1.7. *Let row m be the first row in which all elements of columns 0 to $n-1$ of the Pratt tableau of any real polynomial $P(x) = \sum_{j=0}^{n} c_j x^j$ with $c_0 < 0$ and $c_n > 0$ become negative. Let y be the nth column of the tableau considering the first $(m-1)$ rows. Then if $\delta \geq \frac{-min(y)}{c_n}$, then the real polynomial $P(x) + c_n\delta$ has fewer than 2 roots in the $(0, \infty)$ domain.*

Proof. Considering the real roots of $-P(x)$, the result follows directly from Theorem S1.6. □

Corollary S1.8. *Let row m be the first row in which all elements of columns 0 to $n-1$ of the Pratt tableau of any real polynomial $P'(x) = \sum_{j=0}^{n} c_{n-j} x^j$ with $c_n > 0$ and $c_0 < 0$ become positive. Let y be the nth column of the tableau, considering the first $(m-1)$ rows. Then if $\delta \geq \frac{-max(y)}{c_0}$, then the real polynomial $P(x) = \sum_{j=0}^{n} c_j x^j + c_0\delta$ has fewer than 2 roots in the $(0, \infty)$ domain.*

Proof. As the relevant domain of roots is $(0, \infty)$, considering the real roots of $P'(v)$, $v = \frac{1}{x}$ the result follows directly from Theorem S1.6. □

Theorem S1.9. *For any $\delta^K \geq 0$ there exists δ^P, directly computable from the rate constants, such that if $\frac{\alpha_n^K}{\beta_n^K}[S_{tot}] = \delta_K$ and $\frac{\alpha_0^P}{\beta_0^P}[S_{tot}] \geq \delta^P$ then the polynomial $P'(u)$ has precisely one positive root, corresponding to one steady state. Similarly, for any $\delta^P \geq 0$ there exist a δ_K with the same properties.*

Proof. Comparing Corollary S1.8 with Eq. S9, $c_n = a_{n+1}\left(1 + [S_{\text{tot}}]\frac{\alpha_n^K}{\beta_n^K}\right)$. Setting $\delta^K = [S_{\text{tot}}]\frac{\alpha_n^K}{\beta_n^K} \geq 0$, then $c_n > 0$.

Let $c_0 = a_0$. Then $P'(u)$ in Eq. S9 can be expressed as $P(u) + c_0\delta^P$, where $c_0 < 0$. Then it follows directly from Corollary S1.8 that a $\delta^P = [S_{\text{tot}}]\frac{\alpha_0^P}{\beta_0^P} \geq \frac{-max(y)}{c_0}$ can be calculated with finite summations from the Pratt tableau, which guarantees that there is at most one real positive root. In a real polynomial $P(x) = \sum_{j=0}^{n} c_j x^j$ with $c_n > 0$ and $c_0 < 0$, the number of sign changes has to be odd. From the Descartes' rule of signs the parity of positive real roots and the number of sign changes must be the same. The minimum therefore number of positive roots is 1. Therefore, if the conditions are satisfied there is precisely one positive real root.

□

S1.5 Algorithm for finding δ^K using the Pratt tableau

```
Result: Output δ^K
Input real polynomial P(x) = Σ_{j=0}^{n} c_j x^j with c_0 < 0 and c_n > 0;
Multiply all coefficients c_j by -1;
c_{n+1-j,j} = c_j, 0 ≤ j ≤ n;
k = 1;
while (any c_{k-1,1:n-1} < 0) OR (k - 1 < n) do
    if (k > n) then
        c_{k,0} = c_0 ;
    end
    for j = 1 : n do
        if (k + j) > n + 1 then
            c_{k,j} = c_{k,j-1} + c_{k-1,j}
        end
    end
    k = k + 1;
end
m = max j such that c_{j,n-1} < 0;
y = c_{1:m,n};
δ^K = -max(y) / c_n;
```

S1.6 Algorithm for finding δ^P using the Pratt tableau

Result: Output δ^P
Input real polynomial $P(x) = \sum_{j=0}^{n} a_j x^j$ with $a_0 < 0$ and $a_n > 0$;
Flip coefficients such that $c_{n-j} = a_j$;
$c_{n+1-j,j} = c_j$, $0 \leq j \leq n$;
$k = 1$;
while *(any $c_{k-1,1:n-1} < 0$) OR ($k - 1 < n$)* **do**
  if $(k > n)$ **then**
    $c_{k,0} = c_0$;
  end
  for $j = 1 : n$ **do**
    if $(k + j) > n + 1$ **then**
      $c_{k,j} = c_{k,j-1} + c_{k-1,j}$
    end
  end
  $k = k + 1$;
end
m= max j such that $c_{j,n-1} < 0$;
$y = c_{1:m,n}$;
$\delta^P = \frac{-max(y)}{a_0}$;

S1.7 Generalisation of results

In the general framework of arbitrary processivity and sequentiality, $[S_i] = [S_0]r_i(u)$ [3]. $r_i(u)$ is a rational function of u which was proved to always be well defined and positive in the s-positive sense. A polynomial is said to be sum positive (s-positive) if it is a sum of of positive monomials [3]. A rational function is s-positive if it can be represented as a fraction of two s-positive polynomials.

In this general framework, the three ϕ(u) functions, all being s-positive, are defined as:

$$\begin{aligned} \phi_1(u) =& \sum_{i=0}^{n} r_i(u) \\ \phi_2(u) =& \sum_{i=0}^{n-1} \frac{r_i(u)}{k_i^K} \\ \phi_3(u) =& \sum_{i=1}^{n} \frac{r_i(u)}{k_i^P} \end{aligned} \tag{S10}$$

Therefore, a rational expression in u, $R(u)$, can be defined, where $R(u) = \frac{P(u)}{Q(u)}$. $Q(u)$ is also s-positive.

$$0 = (u - w)\phi_1(u) + [S_{\text{tot}}](u\phi_2(u) - w\phi_3(u)) =: R(u) \tag{S11}$$

where ($w = \frac{[K_{\text{tot}}]}{[P_{\text{tot}}]}$).

From Eq. S10, if all three ϕ(u) functions are expressed with the same least common denominator, which is equal to $Q(u)$, then

$$\begin{aligned} \phi_1(u) &= \frac{P_1(u)}{Q(u)} \\ \phi_2(u) &= \frac{P_2(u)}{Q(u)} \\ \phi_3 &= \frac{P_3(u)}{Q(u)} \end{aligned} \tag{S12}$$

Therefore $P(u)$ can be expressed as:

$$0 = (u - w)P_1(u) + [S_{\text{tot}}](uP_2(u) - wP_3(u)) =: P(u) \tag{S13}$$

Thus, $P(u)$ can be expressed as:

$$P(u) = a_{N+1}u^{N+1} + a_N u^N + ... + a_1 u + a_0 \tag{S14}$$

It is clear from Eq. S10 that order$(P_1(u)) \geq$ order$(P_2(u))$. Similarly, the degree of $P_1(u)$ is no less than the degree of $P_3(u)$. From Eq. S13, it is evident that $a_{N+1} > 0$. Conversely, $a_0 < 0$.

Extending Eq. S14 to include sequestration of the phosphatase by S_0 simply requires adding $\frac{r_0}{k_0^P}$ to ϕ_3 or, equivalently, as $r_0 = 1$ from $[S_i] = [S_0]r_i(u)$, $\frac{1}{k_0^P}Q(u)$ to P_3 in Eq. S13. Thus, $P'(u) = P(u) - [S_{\text{tot}}]w\frac{\alpha_0^P}{\beta_0^P}Q(u)$ with the degree of Q less than that of P. As $\frac{\alpha_0^P}{\beta_0^P}$ is increased, deg(Q) roots of $P'(u)$ tend to the roots of $Q(u)$, none of which are real and positive (since Q has all positive coefficients). The remaining m roots (where $m = \deg(P) - \deg(Q)$) tend to infinity at angles which are multiples of $\frac{2\pi}{m}$ by a standard root locus argument [6]. Thus, for sufficiently large $\frac{\alpha_0^P}{\beta_0^P}$, there will be just one root on the positive real axis.

S1.8 Theoretical development of the new Weakly Chained Diagonally Dominant M-matrix Formulation for the Chemical Master Equation

Row i of a complex matrix A := (a_{ij}) is strictly diagonally dominant (SDD) if $|a_{ii}| > \sum_{i \neq j} |a_{ij}| = \Lambda_i^r(A)$. Similarly, column i of a complex matrix A := (a_{ji}) is strictly diagonally dominant (SDD) if $|a_{ii}| > \sum_{i \neq j} |a_{ji}| = \Lambda_i^c(A)$. A is row/column strictly diagonally dominant (SDD) if all its rows/columns are SDD. Weak diagonal domination (WDD) is defined with weak inequality instead [7]. If not mentioned explicitly, diagonal domination refers to weak row diagonal dominance.

Definition S1.10. [7] A Z-matrix is a real matrix with non-positive off-diagonals.

Definition S1.11. [7] An M-matrix is a monotone Z-matrix (i.e. $Mx \geq 0$ implies that $x \geq 0$).

Definition S1.12. [7] A complex square matrix A is said to be a weakly chained diagonally dominant (WCDD) if it satisfies:

1. A is WDD.
2. for each row r, there exists a path in the graph of A from r to an SDD row p.

Theorem S1.13. *[7, 8] A non-singular WDD Z-matrix with positive diagonals is an M-matrix.*

Theorem S1.14. *[7] The following are equivalent:*

1. *A is a WCDD Z-matrix with positive diagonals.*

2. *A is a WDD M-matrix.*

Proposition S1.15. *[9] Eq. 8 can be formulated into a well-conditioned system of linear equations in the following way:*
$\boldsymbol{A'}q = -A_j$ *where* $\boldsymbol{A'}$ *is the matrix* $\boldsymbol{A}$ *with column* j *removed,* A_j *is the* j^{th} *column of matrix* A *and* q *is a column vector of size* $(n-1)$,
$q = [q_1, q_2, q_3, ..., q_{j-1}, q_{j+1}, q_{j+2}, ..., q_n]^T$, $q_k = \left[\frac{P_s^k}{P_s^j}\right]$.

Karim et al. [9] suggested the formulation shown in Proposition S1.15, which is applicable when the rank of $\boldsymbol{A}$ is equal to $(n-1)$, as a way to translate equation 8 from an ill-conditioned problem into a well-conditioned system of linear equations.

Fact S1.16. *Matrix* $\boldsymbol{A'}$ *of Proposition S1.15 is a rectangular* $n \times (n-1)$ *matrix with rank equal to* $n-1$.

Lemma S1.17. *[10] If X is a zero row sum (ZRS) matrix, then adj(X) has identical rows.*

Corollary S1.18. *If X is a zero column sum (ZCS) matrix, then adj(X) has identical columns.*

Theorem S1.19. *Given any* $i \in \{1, 2, ..., n\}$, *Equation 8 and Proposition S1.15 representing an irreducible Markov process with positive transition rates between distinct states can be further formulated into a solvable format in the following way:*
$\boldsymbol{A}^D_{i,j}q = -A^i_j$ *where* $\boldsymbol{A}^D_{i,j}$ *is the sub-matrix formed after deleting the* i^{th} *row and* j^{th} *column from matrix* $\boldsymbol{A}$ *and* A^i_j *is the the* j^{th} *column of matrix* A_j *as defined in Proposition S1.15 with element* i *deleted.* q *represents the same column vector as in Proposition S1.15.*

Proof. From Fact S1.16, Theorem S1.19 follows if $det(\boldsymbol{A}^D_{i,j}) \neq 0$ and A^i_j is not an empty column vector. A_j must have at least two non-zero elements since by definition $A(\mathbf{x}, \mathbf{x}) = -\sum_{\mathbf{x}' \neq \mathbf{x}} A(\mathbf{x}', \mathbf{x})$ and the process is irreducible, thus strongly connected. Therefore A^i_j has at least one non-zero element. $\boldsymbol{A}$ is a zero column sum (ZCS) matrix, therefore from Corollary S1.18, for a given j, $|det(\boldsymbol{A}^D_{i,j})| = constant$,$i \in \{1, 2, ..., n\}$. It is also known that a column of the adjugate matrix $adj(\mathbf{A})$ is proportional to $\mathbf{P_s}$ [11] (from $\boldsymbol{A}adj(\boldsymbol{A}) = det(\boldsymbol{A}) = 0$ [12]). As the probability of any state found in an irreducible Markov process with positive transition rates between distinct states (which can be represented as a strongly connected graph with positive weights on the edges connecting the different states/nodes) is finite and greater than zero (this is obvious from the the well-known Markov Chain Tree Theorem [10], found here as Theorem S1.30), then for a given j, $|det(\boldsymbol{A}^D_{i,j})| = constant \neq 0$, $i \in \{1, 2, ..., n\}$. □

Corollary S1.20. *State* j *is the unique global mode (state with maximum stationary probability) of the system represented by Equation 8 (representing an irreducible Markov process with positive transition rates between distinct states) if and only if* $\| (\boldsymbol{A}^D_{i,j})^{-1}A^i_j \|_\infty < 1$, *for any* $i \in \{1, 2, ..., n\}$.

Proof. It follows from Theorem S1.19, $q = -(\boldsymbol{A}^D_{i,j})^{-1}A^i_j = [q_1, q_2, ..., q_{j-1}, q_{j+1}, q_{j+2}, ..., q_n]^T$, $q_k = \left[\frac{P_s^k}{P_s^j}\right]$. *For state* j *to be the unique global mode,* $q_k = \left[\frac{P_s^k}{P_s^j}\right] < 1 \forall k \neq j \iff$
$\| q \|_\infty < 1 \iff \| (\boldsymbol{A}^D_{i,j})^{-1}A^i_j \|_\infty < 1$ □

Note that all the matrix norms hereafter are taken to be induced norms.

Corollary S1.21. *Let $\boldsymbol{C} = -\boldsymbol{A}^D_{j,j}$ and $b = A^j_j$ as defined in Theorem S1.19.* $\parallel q \parallel_\infty = \parallel \boldsymbol{C}^{-1} b \parallel_\infty \leq \parallel \boldsymbol{C}^{-1} \parallel_\infty \parallel b \parallel_\infty$

Theorem S1.22. *Let $\boldsymbol{C} = -\boldsymbol{A}^D_{j,j}$ and $b = A^j_j$, as defined in Theorem S1.19. Then* $P^j_s = \frac{1}{1+\|\boldsymbol{C}^{-1}b\|_1} \geq \frac{1}{1+|a_{jj}|\|\boldsymbol{C}^{-T}\|_\infty}$

Proof. $\parallel q \parallel_1 = \parallel \boldsymbol{C}^{-1} b \parallel_1 \leq \parallel \boldsymbol{C}^{-1} \parallel_1 \parallel b \parallel_1 = \parallel \boldsymbol{C}^{-T} \parallel_\infty \parallel b \parallel_1$. But $\parallel q \parallel_1 = \frac{\sum\limits_{k\neq j} P^k_s}{P^j_s} = \frac{1-P^j_s}{P^j_s}$. Therefore, $\frac{1-P^j_s}{P^j_s} \leq \parallel \boldsymbol{C}^{-T} \parallel_\infty \parallel b \parallel_1 \Rightarrow P^j_s \geq \frac{1}{1+\|\boldsymbol{C}^{-T}\|_\infty \|b\|_1} = \frac{1}{1+|a_{jj}|\|\boldsymbol{C}^{-T}\|_\infty}$ □

Fact S1.23. *Matrix $\boldsymbol{C} = -\boldsymbol{A}^D_{j,j}$ as defined in Theorem S1.19 is a square Z-matrix with positive diagonals.*

Fact S1.24. *Matrix $\boldsymbol{C} = -\boldsymbol{A}^D_{j,j}$ as defined in Theorem S1.19 is a column weakly diagonally dominant matrix.*

Theorem S1.25. *Matrix $\boldsymbol{C}^T = -(\boldsymbol{A}^D_{j,j})^T$ as defined in Theorem S1.22 is a weakly chained diagonally dominant (WCDD) M-matrix.*

Proof. From Facts S1.23 and S1.24, $\boldsymbol{C}^T$ is a (row) weakly diagonally dominant (WDD) Z-matrix. From the proof of Theorem S1.19, $\boldsymbol{C}^T$ is nonsingular. From Theorem S1.13, $\boldsymbol{C}^T$ is a WDD M-matrix. Therefore, from Theorem S1.14, $\boldsymbol{C}^T$ is also weakly chained diagonally dominant (WCDD). □

Lemma S1.26. *[13] Assume U is a nonnegative nonsingular matrix. Then, U^{-1} is a row diagonally dominant M-matrix, that is U is a potential, if and only if U satisfies the Complete Maximum Principle (CMP).*

Lemma S1.27. *[13] Assume U is a nonnegative matrix that satisfies the CMP. Then U is column pointwise diagonally dominant. i.e. $\forall i, j\ |U_{jj}| \geq |U_{ij}|$.*

Theorem S1.28. *Let $\boldsymbol{C} = -\boldsymbol{A}^D_{j,j}$ and $b = A^j_j$, as defined in Theorem S1.19. Matrix $\boldsymbol{C}$ is an $m \times m$ matrix, where $m = n-1$. Then* $P^j_s \geq \frac{1}{1+|a_{jj}|trace\left(\boldsymbol{C}^{-T}\right)} = \frac{1}{1+|a_{jj}|\sum\limits_i \lambda_i\left(\boldsymbol{C}^{-T}\right)} = \frac{1}{1+|a_{jj}|\sum\limits_i \frac{1}{\lambda_i(\boldsymbol{C})}} \geq \frac{\lambda_{min}(\boldsymbol{C})}{\lambda_{min}(\boldsymbol{C})+m|a_{jj}|}$. *$\lambda_i(\boldsymbol{C})$ and $\lambda_{min}(\boldsymbol{C})$ denote the ith and the minimum eigenvalue of matrix $\boldsymbol{C}$ respectively.*

Proof. From Lemmas S1.26 and S1.27 and Theorem S1.25, $\parallel \boldsymbol{C}^{-T} \parallel_\infty \leq trace\left(\boldsymbol{C}^{-T}\right) = \sum\limits_i \lambda_i\left(\boldsymbol{C}^{-T}\right) = \sum\limits_i \frac{1}{\lambda_i(\boldsymbol{C})}$. Thus, $P^j_s \geq \frac{1}{1+|a_{jj}|trace\left(\boldsymbol{C}^{-T}\right)} = \frac{1}{1+|a_{jj}|\sum\limits_i \lambda_i\left(\boldsymbol{C}^{-T}\right)} = \frac{1}{1+|a_{jj}|\sum\limits_i \frac{1}{\lambda_i(\boldsymbol{C})}} \geq \frac{\lambda_{min}(\boldsymbol{C})}{\lambda_{min}(\boldsymbol{C})+m|a_{jj}|}$. □

Theorem S1.29. *Let $\boldsymbol{C} = -\boldsymbol{A}^D_{j,j}$ and $b = A^j_j$, as defined in Theorem S1.19. Then* $P^j_s \leq \frac{\sigma_{max}(\boldsymbol{C})}{\sigma_{max}(\boldsymbol{C})+\|b\|_2}$. *$\sigma_{max}(\boldsymbol{C})$ denotes the maximum singular value of matrix C*

Proof. $P^j_s = \frac{1}{1+\|\boldsymbol{C}^{-1}b\|_1} \leq \frac{1}{1+\|\boldsymbol{C}^{-1}b\|_2}$. As $\parallel \boldsymbol{C}^{-1} b \parallel_2 \geq \sigma_{min}\left(\boldsymbol{C}^{-1}\right) \parallel b \parallel_2 = \frac{\|b\|_2}{\sigma_{max}(\boldsymbol{C})}$, $\Rightarrow P^j_s \leq \frac{1}{1+\|\boldsymbol{C}^{-1}b\|_2} \leq \frac{\sigma_{max}(\boldsymbol{C})}{\sigma_{max}(\boldsymbol{C})+\|b\|_2}$ □

S1.9 Relationship with directed spanning trees (arborescences)

[10] Given a directed graph on n vertices, assign weight X_{ij} to the edge from i to j. Given a tree, T, in the directed graph, its weight is given by $W(T) = \prod_{i \to j} X_{ij}$. Theorem S1.30 is the well-known Markov Chain Tree Theorem.

Theorem S1.30. *[10, 14] The ith diagonal of $adj(A)$ is $(-1)^{n-1}$ times the sum of the weights over all directed spanning trees (arborescences) with sink i.*

S1.10 Extension of the stochastic model with the transcription, translation and decay reactions of the substrate and of the enzymes

In the main text, we saw that by controlling the dwell times of particular microstates compared with the spectral properties of the rest of the network, the system can exhibit a bimodal behaviour. Nevertheless, we had considered the system to have a constant number of substrate and enzyme molecules, assuming that those are the total substrate and enzyme numbers at a quasi steady state. It has been shown [15], however, that, given some rate constants, the average concentrations predicted deterministically can be different from what are predicted stochastically.
Therefore, in this section, we investigate whether the system with one available phosphosite with the parameters selected earlier with our stochastic tool, retains its bimodal behaviour when the transcription, translation and decay reactions of the substrate and of the enzymes are also modelled, in the same manner as in [15].

Firstly, for the substrate, the following reactions are modelled.

$$\begin{aligned}
&G_S \xrightarrow{\mathrm{k_1}} G_S + M_S \\
&M_S \xrightarrow{\mathrm{k_2}} M_S + S_0 \\
&M_S \xrightarrow{\mathrm{k_3}} 0 \\
&S_0 \xrightarrow{\mathrm{k_4}} 0 \\
&S_1 \xrightarrow{\mathrm{k_4}} 0
\end{aligned}$$

G_S represents the gene coding for the substrate and M_S represents the mRNA produced. We assume that there is only gene copy in the cell. Similarly for the enzymes, E (representing both phosphatase and kinase),

$$\begin{aligned}
&G_E \xrightarrow{\mathrm{k_5}} G_E + M_E \\
&M_E \xrightarrow{\mathrm{k_6}} M_E + E \\
&M_E \xrightarrow{\mathrm{k_7}} 0 \\
&E \xrightarrow{\mathrm{k_8}} 0
\end{aligned}$$

Simulating the system with the Gillispie Stochastic Simulation Algorithm (SSA), we observe in Fig. C that despite the fluctuations present, the system still exhibits bimodality. The parameters used in the simulation can be found in Section S1.13.

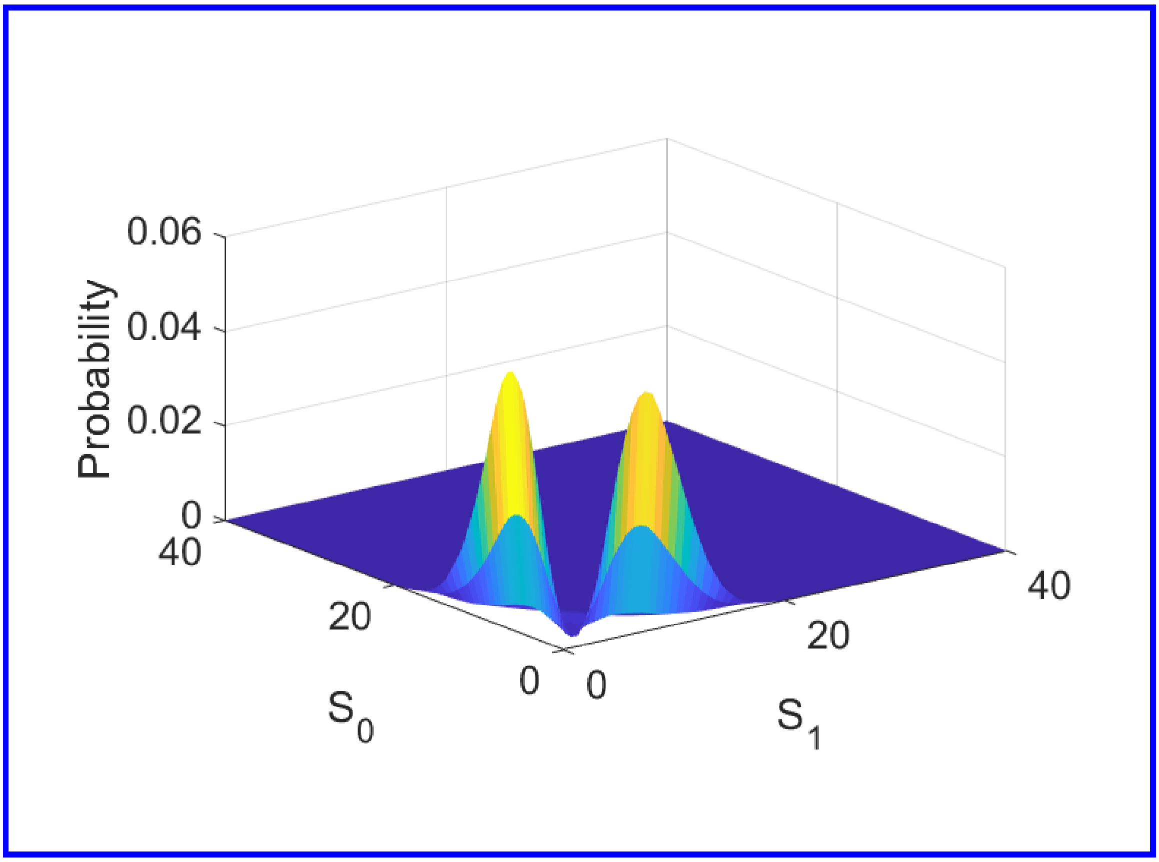

Figure C. By explicitly modeling the transcription, translation and the decay reactions of the substrate and the enzymes, we can see that the system with one phosphosite still exhibits bimodality.

S1.11 Parameters of multisite protein phosphorylation systems under investigation

		$i = 0$	$i = 1$	$i = 2$	$i = 3$
$\alpha_i^K(nM^{-1}sec^{-1})$		8.12×10^{-3}	1.02×10^{-1}	8.12×10^{-3}	1.02×10^{-1}
$\beta_i^K(sec^{-1})$		1.60×10^{-2}	2.04×10^{-1}	1.60×10^{-2}	2.04×10^{-1}
$\gamma_{i,i+1}^K(sec^{-1})$		1.00×10^{-1}	$1.00 \times 10^{+1}$	1.00×10^{-1}	$1.00 \times 10^{+1}$
$\alpha_{i+1}^P(nM^{-1}sec^{-1})$		1.12×10^{-1}	2.64×10^{-3}	6.51×10^{-1}	2.85×10^{-3}
$\beta_{\iota+1}^P(sec^{-1})$		2.24×10^{-1}	5.00×10^{-3}	$1.30 \times 10^{+0}$	6.00×10^{-3}
$\gamma_{i+1,i}^P(sec^{-1})$		$1.10 \times 10^{+1}$	1.70×10^{-2}	$6.39 \times 10^{+1}$	1.36×10^{-1}
$k_i^K(nM)$		$1.43 \times 10^{+1}$	$1.00 \times 10^{+2}$	$1.43 \times 10^{+1}$	$1.00 \times 10^{+2}$
$k_{i+1}^P(nM)$		$1.00 \times 10^{+2}$	$8.33 \times 10^{+0}$	$1.00 \times 10^{+2}$	$5.00 \times 10^{+1}$
λ_ι		6.38×10^{-2}	$5.05 \times 10^{+1}$	1.01×10^{-2}	$3.67 \times 10^{+1}$
$[S_{\text{tot}}](nM)$	$1.00 \times 10^{+4}$				
$[K_{\text{tot}}](nM)$	$2.80 \times 10^{+3}$				
$[P_{\text{tot}}](nM)$	$2.80 \times 10^{+3}$				

Table A. The parameters of the original multisite protein phosphorylation system by Thomson and Gunawardena [1, 3], also used in our analysis

Stochastic Parameter	Stochastic Value	Equivalent Deterministic Parameter	Deterministic Value
Sequestration parameters			
$\alpha(sec^{-1})$	1.0×10^{-2}	$\beta_0^P(sec^{-1})$	1.0×10^{-2}
$\beta(molecule^{-1}sec^{-1})$	1.6×10^{-2}	$\alpha_0^P(nM^{-1}sec^{-1})$	2.4×10^{-5}
$\kappa(molecule^{-1}sec^{-1})$	1.0×10^{-1}	$\alpha_1^K(nM^{-1}sec^{-1})$	1.5×10^{-4}
$\lambda(sec^{-1})$	1.0×10^{-1}	$\beta_1^K(sec^{-1})$	1.0×10^{-1}
Non-sequestration parameters			
$\gamma(molecule^{-1}sec^{-1})$	5.4133×10^0	$\alpha_0^K(nM^{-1}sec^{-1})$	8.12×10^{-3}
$\delta(sec^{-1})$	1.6×10^{-2}	$\beta_0^K(sec^{-1})$	1.6×10^{-2}
$\varepsilon(sec^{-1})$	1.0×10^{-1}	$\gamma_{0,1}^K(sec^{-1})$	1.0×10^{-1}
$\eta(molecule^{-1}sec^{-1})$	$7.47 \times 10^{+1}$	$\alpha_1^P(nM^{-1}sec^{-1})$	1.12×10^{-1}
$\theta(sec^{-1})$	2.24×10^{-1}	$\beta_1^P(sec^{-1})$	2.24×10^{-1}
$\zeta(sec^{-1})$	$1.1 \times 10^{+1}$	$\gamma_{1,0}^P(sec^{-1})$	$1.1 \times 10^{+1}$

Table B. The stochastic parameters used in the single phosphosite system (for a Volume = 2.49×10^{-18} L). The non-sequestration parameters are the same as the ones in the multisite protein phosphorylation system by Thomson and Gunawardena [1, 3]

S1.12 Enzyme-sharing scheme parameters

Parameter	Value	Parameter	Value
$\gamma_s(nM^{-1}sec^{-1})$	1×10^{-5}	$\gamma_z(nM^{-1}sec^{-1})$	1×10^{-3}
$\delta_s(sec^{-1})$	1×10^{-3}	$\delta_z(sec^{-1})$	1×10^{-4}
$\epsilon_s(sec^{-1})$	5×10^{-2}	$\epsilon_z(sec^{-1})$	1×10^{-2}
$\eta_s(nM^{-1}sec^{-1})$	1×10^{-2}	$\eta_z(nM^{-1}sec^{-1})$	1×10^{-1}
$\theta_s(sec^{-1})$	1×10^{-3}	$\theta_z(sec^{-1})$	1×10^{-1}
$\zeta_s(sec^{-1})$	5×10^{-5}	$\zeta_z(sec^{-1})$	2×10^{-4}
$[S_{tot}](nM)$	2×10^{4}	$[P_{tot}](nM)$	3×10^{2}
$[Z_{tot}](nM)$	1.8×10^{4}	$[K_{tot}](nM)$	1×10^{2}

Table C. The parameters for the enzyme sharing scheme of Fig. 8

S1.13 Transcription, translation and decay parameters

Parameter	Value	Parameter	Value
$k_1(sec^{-1})$	15×10^{-3}	$k_2(sec^{-1})$	1×10^{-3}
$k_3(sec^{-1})$	1×10^{-3}	$k_4(sec^{-1})$	2×10^{-3}
$k_5(sec^{-1})$	4×10^{-3}	$k_6(sec^{-1}$	1×10^{-3}
$k_7(sec^{-1})$	1×10^{-3}	$k_8(sec^{-1})$	8×10^{-3}

Table D. The parameters of transcription, translation and decay used in the simulations of Section S1.10

References

1. Thomson M, Gunawardena J. Multi-bit information storage by multisite phosphorylation. arXiv preprint arXiv:07063735. 2007;.
2. Shenolikar S. Protein phosphorylation in health and disease. Academic Press; 2012.
3. Thomson M, Gunawardena J. Unlimited multistability in multisite phosphorylation systems. Nature. 2009;460(7252):274–277.
4. Hardy GH, Littlewood JE, George P. Inequalities. Cambridge University Press; 1991.
5. Pratt JW. Finding how many roots a polynomial has in (0, 1) or (0,∞). American Mathematical Monthly. 1979; p. 630–637.
6. Evans WR. Control system synthesis by root locus method. Transactions of the American Institute of Electrical Engineers. 1950;69(1):66–69.
7. Azimzadeh P, Forsyth PA. Weakly Chained Matrices, Policy Iteration, and Impulse Control. SIAM Journal on Numerical Analysis. 2016;54(3):1341–1364.
8. Plemmons RJ. M-matrix characterizations. I-nonsingular M-matrices. Linear Algebra and its Applications. 1977;18(2):175–188.
9. Karim S, Buzzard GT, Umulis DM. Efficient calculation of steady state probability distribution for stochastic biochemical reaction network. BMC genomics. 2012;13(Suppl 6):S10.
10. Feinsilver P. Matrices with zero row sums, tree theorems and a Markov chain on trees. Probability on Algebraic and Geometric Structures. 2016;668:67.
11. Basile R, Grima R, Popović N. A graph-based approach for the approximate solution of the chemical master equation. Bulletin of mathematical biology. 2013;75(10):1653–1696.
12. Press WH, Dyson FJ. Iterated Prisoner's Dilemma contains strategies that dominate any evolutionary opponent. Proceedings of the National Academy of Sciences. 2012;109(26):10409–10413.
13. Dellacherie C, Martinez S, San Martin J. Inverse M-matrices and ultrametric matrices. vol. 2118. Springer; 2014.
14. Anantharam V, Tsoucas P. A proof of the Markov chain tree theorem. Statistics & Probability Letters. 1989;8(2):189–192.
15. Grima R, Walter NG, Schnell S. Single-molecule enzymology à la Michaelis–Menten. The FEBS journal. 2014;281(2):518–530.
